# A Swedish genome-wide haplotype association analysis identifies novel candidate loci associated with endometrial cancer risk

**DOI:** 10.1371/journal.pone.0316086

**Published:** 2025-03-18

**Authors:** Elin Barnekow, Wen Liu, Emil Andersson, Xuemin Wang, Hafdis T. Helgadottir, Jessada Thutkawkorapin, Serena Barilla, Litika Vermani, Miriam Mints, Emma Tham, Peter A. Fasching, Diether Lambrechts, Frédéric Amant, Amanda B. Spurdle, Per Hall, Tracy A. O’Mara, Sara Margolin, Annika Lindblom

**Affiliations:** 1 Department of Clinical Science and Education, Södersjukhuset, Karolinska Institutet, Stockholm, Sweden; 2 Department of Oncology, Södersjukhuset, Stockholm, Sweden; 3 Department of Molecular Medicine and Surgery, Karolinska Institutet, Stockholm, Sweden; 4 Department of Surgical Sciences, Uppsala University, Uppsala, Sweden; 5 Department of Women´s and Children’s Health, Karolinska University Hospital, Stockholm, Sweden; 6 Cancer Research Program, QIMR Berghofer Medical Research Institute, Brisbane, Queensland, Australia; 7 Department of Clinical Genetics, Karolinska University Hospital, Stockholm, Sweden; 8 Department of Computer Engineering, Faculty of Engineering, Chulalongkorn University, Bangkok, Thailand; 9 Department of Gynecology and Obstetrics, University Hospital Erlangen, Erlangen, Germany; 10 Comprehensive Cancer Center Erlangen-EMN, Friedrich-Alexander University Erlangen-Nuremberg, Erlangen, Germany; 11 Department of Human Genetics, VIB Center for Cancer Biology, University of Leuven (KU Leuven), Leuven, Belgium; 12 Division Gynecologic Oncology, UZ Leuven, Leuven, Belgium; 13 Population Health Program, QIMR Berghofer Medical Research Institute, Brisbane, Queensland, Australia; 14 Department of Medical Epidemiology and Biostatistics, Karolinska Institutet, Stockholm, Sweden; University of Iceland, ICELAND

## Abstract

Genome-wide association studies [GWAS] have identified a limited number of endometrial cancer risk loci by analyzing single nucleotide polymorphisms [SNPs]. We hypothesized that analyzing haplotypes rather than SNPs could provide novel and more detailed information on genetic cancer susceptibility loci. To examine the association of a SNP or haplotype with endometrial cancer risk we performed a two-stage haplotype GWAS. The discovery GWAS included a sub-cohort of 1,116 Swedish endometrial cancer cases and 5,021 controls from previously published GWAS data. A sliding window analysis was employed with window sizes of 1-25 SNPs using a logistic regression model. The Swedish haplotype analysis identified 15 novel candidate risk loci (2q31.1, 4p16.1, 4p15.31, 6q13, 7p21.1, 9p13.3, 10q26.3, 11q21, 12q13.11, 13q12.11, 15q13.3, 16q24.3, 19q13.32, 20p12.3 and 22q13.2) with OR ranging from 1.6 to 3.3 and p-values from 4.25 × 10^−8^ to 9.86 × 10^−15^. A second replication haplotype analysis of the Swedish novel loci was performed using two cohorts from Belgium and Germany. In spite of small sample sizes in the replication cohorts, there was still support for most loci with positive ORs. In addition, the findings in the two European cohorts motivates further studies to search for founder haplotypes. These novel findings suggested that endometrial cancer loci, identified through haplotype analysis, conferred a higher risk compared to previous single-variant GWAS.

## Introduction

Endometrial cancer [EC] is the sixth most common cancer in women worldwide and the fourth most common cancer in women in high-income countries, with an increasing incidence in recent years [1]. There are well-known life-style risk factors that contribute to EC development and increase in incidence [2]. There are also well-known inherited genetic factors that contribute significantly to EC risk [3]. EC risk is especially high in women with inherited loss-of-function variants in specific genes [3]. Lynch syndrome is the most common high-risk EC syndrome, responsible for approximately 2-5% of all EC cases [3]. After excluding known EC germline variant carriers, the relative risk of EC in first-degree relatives is two-fold compared to the general female population [4]. This indicates that unexplained heritable factors are involved in EC susceptibility. Recent genome-wide association studies [GWAS] of single nucleotide polymorphisms [SNPs] have identified several low-risk loci for EC, however these explain less than 10% of the familial risk of EC [5–7].

Haplotypes are a specific consecutive combination of SNPs located close to each other on a chromosome and are usually preserved through many generations because they are rarely affected by larger relocations of the genome during homologous recombination cycles. Studying haplotypes instead of individual SNPs in cancer susceptibility and other complex diseases could increase the power of statistical analysis and could define a risk locus more precisely, as shown previously [8–10]. Thus, identified unique risk haplotypes define the borders of a DNA region holding an assumed risk variant between the first and last marker.

We hypothesized that haplotype analysis could be more powerful than SNP-only-analysis in identifying novel EC susceptibility loci with higher risk. To test this hypothesis, we conducted a two stage GWAS in EC cases and controls. Previous GWAS on EC risk have been performed in heterogeneous populations [5–7]. Since Sweden has a more homogenous population, making it more beneficial for haplotype GWAS, we conducted the discovery GWAS using only Swedish cases and controls from the Endometrial Cancer Association Consortium [ECAC] study [5]. For validation, a replication GWAS of the Swedish novel loci were performed in two small European cohorts from genetically similar populations (Belgian and German) together with the same Swedish cohort to enable haplotype comparison and to identify potential founder mutations [5].

## Materials and methods

### Discovery GWAS

#### Study population.

Two Swedish cohorts of invasive EC cases were included in this study. Patients within the first cohort, RENDOCAS (n = 555), underwent surgery for EC at the Karolinska University Hospital between 2008 and 2011 [11]. Patients within the second cohort, CAHRES (n = 578), were postmenopausal women diagnosed with EC in Sweden between January 1994 and December 1995 [12]. A total of 5,032 healthy Swedish controls were recruited from the KARMA cohort, consisting of women aged 40-74 years who underwent mammographic screening at four hospitals in Sweden between January 2011 and March 2013 [10,13]. The studies were approved by the local ethical board and all study participants gave written informed consent (KARMA: Approved by the Ethical Committee of the Karolinska Institute, 2010/958-31/1; RENDOCAS 2010/1536-31/2; CAHRES 98-036)

#### Genotyping and quality control.

Genotyping was performed using the custom-designed OncoArray-Chip (Illumina OncoArray 534K) within the collaboration of ECAC and Breast Cancer Association Consortium, BCAC [5,14,15]. The authors had no access to information that could identify individual participants from the genotype data provided by ECAC and BCAC. The genotype data from KARMA was accessed 8 September 2017 for research purposes, RENDOCAS and CAHRES 21 September 2017. The Swedish cohorts shared 474,706 SNPs. Data were merged using PLINK v 1.9 [16]. A total of 6,165 individuals (1,133 cases and 5,032 controls) underwent Quality Control [QC] analysis. SNPs with a call rate < 98%, minor allele frequency [MAF] < 1% and those with genotype distribution inconsistent with Hardy-Weinberg equilibrium (p < 0.001) were removed. In total 141,800 SNPs failed to meet the QC criteria.

In the final QC step, multidimensional scaling [MDS] analysis was conducted on the remaining markers and individuals to identify genetic ethnic outliers and for population stratification purpose [17]. The scaling was based on the predifined mds coordinates 1 (C1), 2 (C2), 3 (C3), and 4 (C4), which represented the position in four dimensions. The value of the coordinates represents the distances from the center of the graph using proximity scaling. Plotting individuals based on their values in four dimensions provides a scatter plot. In our study, we chose dimension limits from −0.04 to 0.04. Individuals outside the limits were considered genetic ethnic outliers and were excluded from the dataset (n = 17 EC cases and 11 controls). The remaining individuals were plotted in a MDS plot (S1 Fig). After QC, 332,906 SNPs remained to perform further downstream analyses. In total, 1,116 EC cases and 5,021 KARMA controls remained for analysis.

#### Genome-wide haplotype analysis.

A sliding window haplotype GWAS was performed using PLINK v1.07, from a window size of 1 to 25 SNPs, with a logistic regression model to examine the association between SNPs and haplotypes of various lengths and EC risk. The default setting of minor haplotype frequency 0.01 in PLINK v.1.07 was applied. This entails that each haplotype or SNP with a frequency above 1% was tested individually against all other SNPs/haplotypes with a frequencies above this threshold [16,18]. Corresponding odds ratio [OR], 95% confidence intervals, and p-values were calculated using the default settings of haplotype analysis in PLINK v1.07 [16]. To adjust for population stratification, C1-C4 (see “Genotyping and quality control”) were used as covariates in the logistic regression model. For SNP analysis, the genomic inflation factor was calculated (λ =  1.03). No adjustments were performed for age. The effect of exposure on outcome was estimated using the OR. The reference genome panel GRCh37 was applied. The purpose of this study was to identify candidate risk loci associated with EC, therefore only loci with an OR > 1 were reported in this study. Genome-wide significance (p-value <  5 × 10^ − 8^) was considered statistically significant [19]. No correction for multiple testing was performed because we assumed that all haplotypes of various length in each sublocus reflected the same genetic risk locus. A quantile-quantile [QQ] plot was created to illustrate the observed association of SNPs with EC compared with the expected null distribution (S2 Fig). A Manhattan plot was created to display the observed P-values along the chromosomes for haplotype- (S3 Fig) and SNP associations (S4 Fig). QQ- and Manhattan plots were generated in R using the qqman package.

### Replication GWAS

####  Study population.

We conducted replication analysis of the 15 loci detected in the discovery GWAS in two separate cohorts (Belgian and German), previously used within the collaboration of ECAC [5]. Replication in the Belgian population included 528 cases from the Leuven Endometrial Cancer Study (LES) and 1,266 controls from blood donors of the Leuven Multidisciplinary Breast Centre Study (LMBC). Replication analysis in the German population included 221 cases from the Bavarian Endometrial Cancer Study (BECS) and 251 healthy controls aged 55 years and older from the Bavarian Breast Cancer Cases and Controls (BBCC). The same Swedish cohort from the discovery analysis was used in the replication stage, but with 6 more cases and 1,059 more controls included (1,139 cases and 6,080 controls). The studies were approved by the local ethical board and all study participants gave written informed consent (LES & LMBC S57278 - MLI 1158; BECS&BBCC Nr 2700).

#### Genotyping and quality control.

Belgian and German samples were genotyped using the same platform as the Swedish discovery samples, i.e., the Illumina OncoArray 534K platform [5,15]. The genotype data from LES, LMBC, BECS, and BBCC was accessed 21 April 2023. To ensure comparability between the Swedish, German, and Belgian cohorts, we conducted QCs using the combined samples. This resulted in a total of 9,485 individuals (1,888 cases and 7,597 controls) undergoing the combined QCs. A common set of 483,972 SNPs were genotyped for the Swedish, Belgian, and German cohorts. The data were merged, and the TOP strand format was accounted for using PLINK v 1.07 [16]. Identical QC criteria (excluding variants with call rate < 98%, MAF < 1%, HWE, p < 0.001, or missing genotypes for samples <  0.1) and MDS exclusion criteria (<−0.04 or > 0.04) were used in the discovery and replication set. After the combined QCs, 399,591 variants remained for analysis (752 variants were excluded by HWE p <  0.001, 1,791 variants failed the missingness test (GENO >  0.02), 82,276 variants failed the frequency test), and no sample was excluded during the QC process.

#### Replication, haplotype analysis of 15 loci.

Targeted haplotype analysis of the 15 novel loci was performed separately in the Belgian, German, and Swedish cohorts. To examine the association between haplotypes and EC in these regions a sliding window analysis, from window size 1 to 25 SNPs was performed with a logistic regression model including C1-C4 from MDS analysis as covariates. Corresponding OR and p-values were calculated accordingly using the default settings of haplotype analysis in PLINK v1.07 [16]. Because of the limited size of the Belgian and German cohorts, we did not expect statistically significant values generally used for GWAS in replication. Instead, we focused on the odds ratios and present the p-values from the PLINK analysis without correction for multiple testing across the 15 loci.

##  Results 

To examine the association between a specific combination of close SNPs (a haplotype) and EC risk a two stage GWAS was performed. In the discovery Swedish dataset of 1,116 EC cases and 5,021 controls 332,906 SNPs were analyzed which resulted in 8,315,750 sliding windows. Significant SNPs or haplotypes in a delimited region is defined as one susceptibility locus and typically represented by several haplotypes of different lengths, ORs and statistical significance. The discovery haplotype GWAS identified 15 novel susceptibility loci located on chromosomes 2q31.1, 4p16.1, 4p15.31, 6q13, 7p21.1, 9p13.3, 10q26.3, 11q21, 12q13.11, 13q12.11, 15q13.3, 16q24.3, 19q13.32, 20p12.3 and 22q13.2 (Table 1, S1–23 Tables, S3 and S4 Figs). The loci had a frequency ranging from 1% to 8%, ORs from 1.6 to 3.3 and p-values from 4.25x10^−8^ to 9.86x10^−15^ (Table 1). Three loci spanned more than one gene, 11 loci spanned one gene, and one locus spanned no known gene (Table 1). The detailed haplotypes for each locus are listed in S24 Table. One significant SNP was identified on 16q24.3 (OR 1.58, p 3.36x10^−8^) (Table 1, S25 Table and S4 Fig). For the other novel loci, the lowest p-value for one SNP ranged from 3.8x10^−6^ to 0.19 (S25 Table).

**Table 1:** Haplotypes with lowest P value in 15 endometrial cancer risk loci.

Locus	Gene	Haplotype	SNP1- SNP2 (BP1-BP2)	F	OR (95% CI)	P-value
2q31.1	*ITGA6*	GAAGGTG	rs12053442-rs36055280 (173295405-173296850)	0.03	2.15 (1.70-2.72)	1.53 x 10^−10^
4p16.1	*KIAA0232*	GGGAGGG	rs6833118-rs7675928 (6833794-6890937)	0.03	2.08 (1.61-2.69)	1.92 x 10^−8^
4p15.31	*KCNIP4*	ACGGAAGGAAGATCAGGGCGACG	rs10938823-rs1156764 (21007589-21208312)	0.01	2.7 (1.90-3.84)	2.96 x 10^−8^
6q13	*KCNQ5*	ACAGAAAAACGGGGG	rs1572208-rs6913237 (73171594-73343796)	0.02	2.19 (1.65-2.90)	4.25 x 10^−8^
7p21.1	*ABCB5*	AAGGGGG	rs966717-rs34908430 (20796585-20824614)	0.02	2.04 (1.59-2.62)	2.37 x 10^−8^
9p13.3	*RUSC2, FAM166B, CD72,* *TESK1, SIT1, RMRP*	GAAGCAGGGGAAGAA	rs10972503-rs3750430 (35532053-35658163)	0.03	1.99 (1.56-2.54)	3.94 x 10^−8^
10q26.3	*TTC40*	AGAGAAA	rs913191-rs7907613 (134615651-134705373)	0.02	3.05 (2.13-4.37)	1.32 x 10^−9^
11q21	*HEPHL1, PANX1, FOLR4*	GGGCACGACGGGGACAAAGGAAAC	rs4753116-rs484889 (93767360-94078269)	0.01	2.63 (1.86-3.71)	3.78 x 10^−8^
12q13.11	*–*	GAGAGGAACCAGGGC	rs7967938-rs2166138 (46439995-46537244)	0.01	3.17 (2.13-4.71)	1.07 x 10^−8^
13q12.11	*TUBA3C*	AGGAGAGAGAAGA	rs17090505-rs7338881 (19690302-19761203)	0.01	3.31 (2.28-4.80)	2.90 x 10^−10^
15q13.3	*GREM1*	GAAAAGAAGAAAAAAAGGTGGAC	rs55659128-rs8034965 (32996214-33011851)	0.02	2.2 (1.70-2.84)	1.43 x 10^−9^
16q24.3	*ANKRD11*	G	rs12928649 (89333342)	0.08	1.58 (1.34-1.86)	3.36 x 10^−8^
19q13.32	*PVRL2, TOMM40, APOE*	AAAGAGA	rs11669338-rs7412 (45382984-45412079)	0.02	2.51 (1.88-3.35)	4.34 x 10^−10^
20p12.3	*TMX4*	AGAAAGCGGCGAC	rs4813847-rs6055481 (7966974-8009127)	0.03	2.42 (1.93-3.03)	9.86 x 10^−15^
22q13.2	*SCUBE1*	GGGCGGCGAAAGAAAAGAAGG	rs5996306-rs695648 (43689984-43760187)	0.02	2.9 (2.02-4.15)	6.55 x 10^−9^

Each locus presented with gene in the area (if any), haplotype (window size 1-25), first (SNP1) and last SNP (SNP2), first (BP1) and last (BP2) genomic position, corresponding frequency(F), odds ratio (OR), and p-value (P) for haplotypes of window size 1-25. Reference panel GRCh37.

All suggested 15 susceptibility loci were novel, and validation was therefore warranted. Two European cohorts were used for replication. Theoretically, a haplotype defines the borders of a susceptibility region that contains a possible underlying mutation. Therefore, we aimed to replicate the 15 haplotype regions from the discovery GWAS (Table 1) from the first to the last SNP at each locus. The significant SNP at 16q24.3 was sorted out during the QC for the replication analysis. Therefore the haplotype with a SNP before and after is presented for comparison in the replication analysis. To identify potential founder mutations identical SNPs are required for comparison. This was assured with a new QC with combined samples from the Swedish, Belgian and German cohorts. After QC, a separate haplotype analysis with a window size of 1-25 was performed in the two replication cohorts and the enlarged Swedish cohort (with 6 new cases and 1,059 new controls).

**Fig 1:**
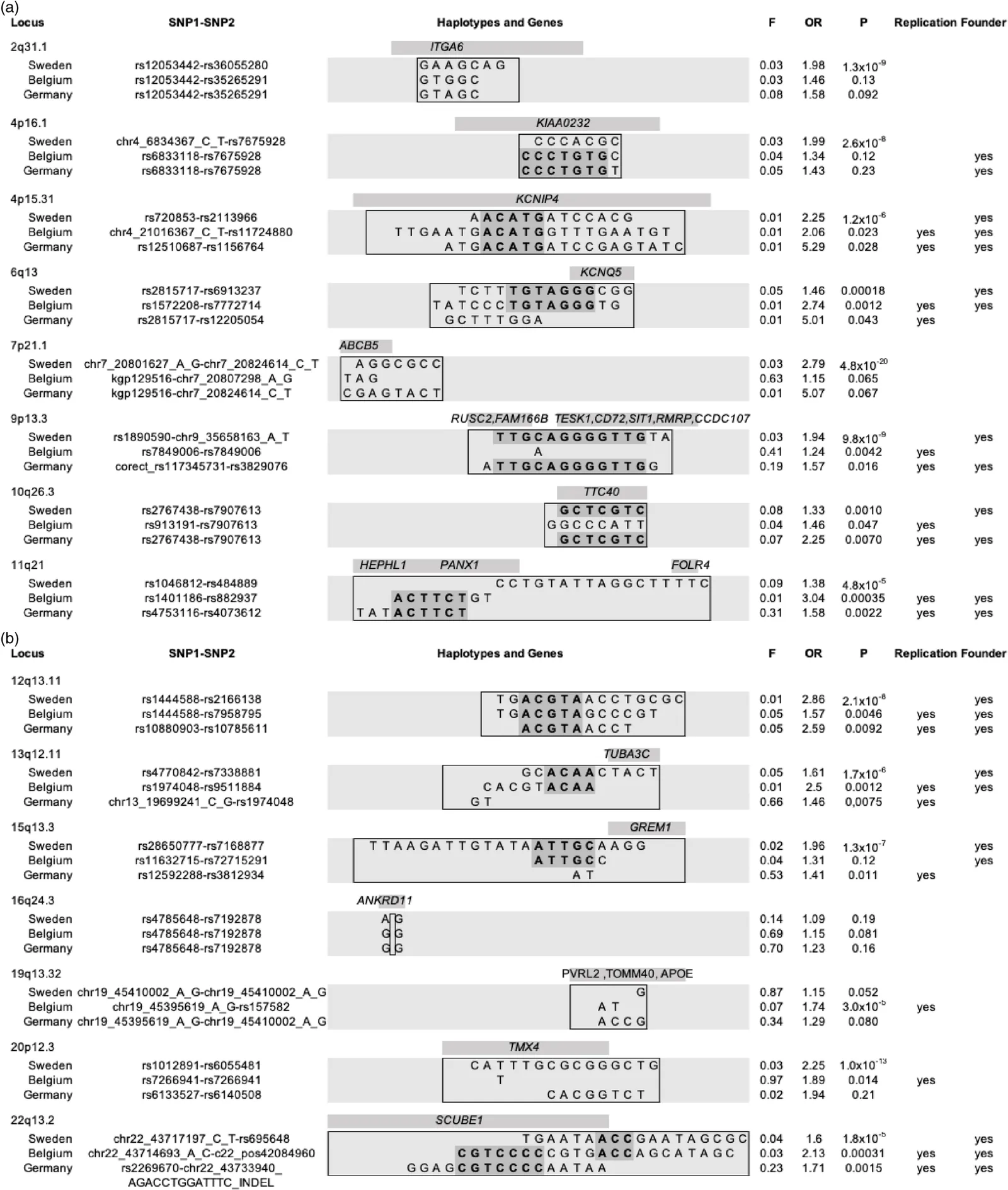
Replication of the 15 novel endometrial cancer risk loci. Replication GWAS in a German (221 cases; 251 controls) and a Belgian cohort (528 cases; 1,266 controls) along with the Swedish cohort (1,139 cases; 6,080 controls). Each locus presented with first (SNP1) and last SNP (SNP2), genes in the area (if any). The haplotype with the lowest p-value in each cohort is presented within the discovery locus (marked with a box) with corresponding frequency (F), odds ratio (OR), p-value (P) and indication of replication (P < 0.05). Shared identical haplotypes between the cohorts are marked in dark grey, indicating possible founder variants. The discovery SNP on 16q24.3 (marked with a box) was not analysed in the replication analysis but situated between the two SNPs in the replication haplotype.

A locus was considered replicated if the replication GWAS presented any SNP or haplotype in the discovery region with a p-value <  0.05. Eight of the novel Swedish loci from the discovery GWAS were replicated in both the German and Belgian cohorts (4p15.31, 6q13, 9p13.3, 10q26.3, 11q21, 12q13.11, 13q12.11 and 22q13.2); whereas two loci (19q13.32 and 20p12.3) were replicated in the Belgian cohort and one locus (15q13.3) was replicated in the German cohort (Fig 1) (S26 Table). The loci at 2q31.1, 4p16.1, 7p21.1 and 16q24.3 were not replicated in either cohort.

Shared identical haplotypes between cohorts may indicate a possible underlying founder mutation. Possible European founders were suggested on 4p15.31 and 12q13.11, where identical haplotypes between the Swedish, German, and Belgian cohorts were identified. Two possible founders were suggested on 22q13.2, one Swedish-Belgian and one Belgian-German. An indication of a Swedish-Belgian founder was seen on 6q13, 13q12.11 and 15q13.3, a Swedish-German founder on 9p13.3 and 10q26.3, and a Belgian-German founder on 4p16.1 and 11q21 (Fig 1).

## Discussion

In the search for founder haplotypes of endometrial cancer, we reanalyzed genotype data from Swedish samples included in ECAC by performing a discovery haplotype GWAS of 1,116 invasive EC cases and 5,021 controls. This haplotype GWAS suggested 15 novel risk loci for EC, of which eight were replicated in two separate small European cohorts and an additional three in any of the two cohorts. Each haplotype conferred a two- to three-fold increased risk of EC. Our study demonstrated that haplotype analysis was able to identify novel risk loci with higher ORs than previous GWAS based on single variants [5–7].

Two European cohorts were available for validation analysis. Both were however small, which limited the ability to achieve statistical significance in this analysis. Nonetheless, the consistent presence of positive ORs for the haplotypes, similar to those observed in the discovery analysis, indicated that risk alleles are likely located within these haplotypes and even suggested the possibility of European founders. Identification of potential founder mutations required a new QC including all the three cohorts to involve identical SNPs for comparison. This means that the Swedish replication result was not identical to the discovery result. This was expected, as GWAS is highly dependent on the markers used in the analysis. Importantly, all loci had positive ORs in the replication for all three cohorts. All loci which showed compelling associations in the Swedish discovery cohort did not reach the same level of statistical significance in the replication sets. Nevertheless, this could be explained by discrepancies between markers in the discovery and the replication analyses. Using different markers in two GWAS conducted on the same data set may not identify the same haplotypes. Moreover, due to genetic population heterogeneity between the Swedish, German and Belgian cohorts, we cannot expect all loci to be replicated.

We have previously demonstrated the ability to use haplotype analysis, in addition to SNP analysis, when identifying novel risk loci for cancer in general as well as for colorectal and breast cancer in a Swedish population [8–10]. We suggest that haplotype analysis may define the spanned locations for disease-causing variants. SNP analysis results in an exact position for a risk-associated SNP; however, the distance to the actual disease-associated variant in SNP analysis is often unknown. In contrast, a risk haplotype is assumed to define the borders of the region containing the disease-causing variant. However, more homogenous populations are required to enable this strategy to identify founder risk haplotypes. Further genotyping studies could search for founder haplotypes.

Haplotype analysis also demonstrated the complexity of the genetic contribution of low- to moderate-risk loci, depending on the combined effect of more than one disease-causing variant. This was supported by the present study, in which three risk haplotypes covered more than one gene (Table 1), a finding that was also seen in our previous studies [8–10]. The odds ratios for the risk haplotypes were higher than those previously reported in published SNP GWAS [5–7]. The higher ORs could be explained by the superior ability of a haplotype to identify a population at risk, compared to a single variant. Therefore, haplotype analysis resulted in higher ORs than single variant analysis at the same locus (Table 1 and S26 Table). This is consistent with our previous findings from haplotype GWAS [8–10]. Single variant analysis showed similar ORs and frequencies of known EC susceptibility SNP´s in the Swedish and previous ECAC analyses for the SNP´s included in the present analysis. However, a statistically significant level was not reached, probably because of the smaller sample size (S27 Table) [5–7].

The P-value criteria for a sliding window haplotype analysis are complicated and can be discussed as multiple testing is performed. Numerous overlapping haplotypes were generated in the sliding window analysis. We assumed that the various haplotypes in each delimited region (S1–S23 Tables) reflect the same genetic risk and thus could represent one risk locus. Therefore, no correction for multiple testing was performed and p < 5x10^−8^ was used for the discovery analysis. The cohorts used for replication in this study were small and further studies are required to replicate the Swedish results in other European populations.

Haplotype analysis revealed one locus on chromosome 16. The haplotype with the lowest p-value in this region was a single variant, suggesting that the actual risk variant may be very close to this variant (Table 1). In the replication analysis, this SNP was not analyzed, as it was sorted out during the QC of the replication sets. There are three overlapping elements in this small region: the gene *ANKRD11*, a coding protein *AC137932.1-201* and a ribosomal DNA, *RP11-46C24.5. ANKRD11* is a chromatin regulator and *p53* activator that has been studied in several cancer types and has recently been suggested to be involved in predisposition to ovarian cancer [20,21].

Several genes located in these 15 loci have been previously discussed in relation to cancer, although many have not been previously reported to be involved in EC. *GREM1,* located at the novel locus 15q13.3, is known for its role in hereditary mixed colorectal cancer syndrome [22]. The potassium channel-interacting protein 4 gene *(KCNIP4)* at 4p15.31 has been proposed as a candidate gene for renal cell carcinoma and was recently identified as a lung cancer risk gene in a familial GWAS [23,24]. The potassium channel voltage-gated KQT-like subfamily member 5 *(KCNQ5)* at 6q13 is considered a hypermethylation marker in colorectal cancer [25]. The ATP-binding cassette member 5 gene *(ABCB5)* at 7p21.1, which is upregulated in cancers, such as skin-, breast-, colon cancer and melanoma, is a multidrug resistance mediator in diverse malignancies [26,27]*. TTC40* at 10q26.3 was found to be amplified in HER2-negative gastric cancer in one study and a nearby suggested tumor suppressor gene at locus 10q26.1, the cancer susceptibility candidate 2 gene (*CASC2*), is commonly lost in EC [28,29]. The locus on 12q13.11 holds no gene but is located close to *SLC38A1*, which has been studied in many types of cancer and specifically suggested to have a role in EC, where an upregulation of the gene was observed and a downregulation of the gene resulted in decreased EC cell proliferation [30]. *TMX4* at 20p12.3 promotes palmitoylation of cysteine residues adjacent to the membrane-spanning domain of the transmembrane thioredoxin family (TMX) [31]. Palmitoylation is essential for proteins encoded by both oncogenes and tumor suppressor genes. Therefore, activation of *TMX4* may be a risk factor for cancer [32]. The locus on 22q13.2 showed two possible founder variants, one Swedish-Belgian and one German-Belgian. The Belgian haplotype, including both founder regions presented higher OR than the single founder Swedish and German haplotypes, consistent with an increased risk resulting from the two variants. The signal peptide-, cub domain- and EGF-like domains-containing protein 1 (*SCUBE1*) at the 22q13.2 locus is a promising biomarker in renal cancer, and possibly also in breast cancer [33,34].

Some genes involved in the 15 novel EC loci have been shown to play a role in the tumor microenvironment. Integrin gene alpha-6 *(ITGA6)* at locus 2q31.1 has been linked to a more metastatic cell phenotype in cancer [35]. Integrins play an important role in cell adhesion through cooperation with different cell surface receptors, including growth factor and G protein-coupled receptors [36]. Many studies have provided evidence for the role of laminin-binding integrins in tumorigenesis, and both tumor-promoting and tumor-suppressive activities have been identified [37].

A genome-wide association with a trait can be either a direct genetic effect or an indirect effect through an associated risk factor. Interestingly, the gene *TUBA3C* at 13q12.11 has been suggested to play a role in obesity, which is a known risk factor for EC [38]. *KIAA0232,* at 4p16.1*,* has been associated with platelet counts *[*39*],* and has been reported to affect cancer development and progression [40].

Three loci suggest haplotypes spanning more than one gene. The 9p13.3 locus spans six genes. A Swedish-German founder haplotype confirmed this locus, which was further supported by a SNP in the Belgian cohort. It is possible, however unlikely, that all genes contribute to a risk variant of this haplotype. Nevertheless, the large Swedish and German haplotype in this region indicated that more than one risk variant could be involved. *RUSC2* is associated with cancer therapy pathways, indicating its role in cancer development [41]. Other genes have also been reported to be involved in cancer. *Fam166B* has been found to be upregulated in adipose tissue, possibly with an effect on cancer risk [42]. *CD72* is constantly expressed in chronic lymphocytic leukemia and other B-cell lymphoproliferative disorders [43]. *TESK1* has been suggested to be involved in cancer development [44]. *SIT1* has been implicated in B-cell proliferation [45], and *RMRP* has been suggested to have an oncogenic role in hepatocellular carcinoma [46]. In the locus on 11q21 three possible genes may be involved. *HEPHL1* and *PANX1* were proposed to have a founder mutation in Belgium and Germany, while the Swedish analysis suggested only an association with *FOLR4*. It is still possible that the other variants were missed in the replication analysis depending on the other SNPs used in the analysis. Both *HEPHL1* and *PANX1* have been previously described in relation to cancer, although this is the first implication in EC. Three SNPs of *HEPHL1* are associated with dietary iron intake and colorectal adenoma, and *PANX1* appears to facilitate tumor growth and metastasis in tumor cells [47,48]. Finally, the locus on 19q13.32 presented a haplotype including three genes, *PVRL2, TOMM40* and *APOE* in the first analysis. In the replication, both the German and Belgian analysis resulted in OR > 1, but p-value below 0.05 only in the Belgian analysis. In a recent study, co-expression of *PVRL2* was observed in multiple tumor types, interestingly with the highest co-expression observed in EC [49]. *APOE* has also been suggested to be associated with EC, and increased *APOE* expression has been associated with poorly differentiated endometrial tumors [50,51]. *TOMM40* overlaps the *APOE*.

## Conclusion

In conclusion, a discovery haplotype analysis identified 15 novel candidate loci associated with increased EC risk in a Swedish cohort. A second replication study supported most of the loci with positive ORs and presented identical genotypes between populations at some loci. These results support the hypothesis that haplotype GWAS could be powerful in identifying risk loci with higher risks than previous GWAS. The results suggested that more than one risk gene/variant could contribute to the cancer risk at one locus. Further studies are warranted to replicate this study and how to incorporate the results in clinical risk prediction.

## Supporting information

S1 Fig MDS plot, individuals after excluding ethnic outliers. This is the S1 Fig legend. Each dot is an individual (case or control). The value of coordinate 1 (C1), C2 (blue), C3 (orange) and C4 (grey) represent the distance from the center of the graph in four dimensions representing genetic ethnicity based on the variants included in the analysis.
(PDF)

S2 Fig Quantile-quantile plot of SNPs in Swedish endometrial cancer. This is the S1 Fig legend. Black dots represent observations scaled down by an inflation factor of 1.03. The diagonal red line represents the expected null hypothesis ( = no association).
(PDF)

S3 Fig Haplotype Manhattan plots. This is the S1 Fig legend. Manhattan-plot of haplotype association in 15 novel endometrial cancer candidate risk loci.
(PDF)

S4 Fig SNP Manhattan plot. This is the S1 Fig legend. Manhattan-plot of SNP association in Swedish endometrial cancer. Observed p-values for SNP association on the y-axis. Autosomal chromosome numbers on the x-axis. The horizontal red line represents p < 5x10^ − 8^. Statistically significant SNP rs12928649 is presented.
(PDF)

S1 Table Association SNP-/haplotype and endometrial cancer, chromosome 1. Associations between SNP-/haplotype and endometrial cancer, Chr 1 sorted on p-value. Due to the excel-sheet limitation of rows the complete data is not included.
(XLSX)

S2 Table Association SNP-/haplotype and endometrial cancer, chromosome 2. Associations between SNP-/haplotype and endometrial cancer, Chr 2 sorted on p-value. Due to the excel-sheet limitation of rows the complete data is not included.
(XLSX)

S3 Table Association SNP-/haplotype and endometrial cancer, chromosome 3. Associations between SNP-/haplotype and endometrial cancer, Chr 3 sorted on p-value. Due to the excel-sheet limitation of rows the complete data is not included.
(XLSX)

S4 Table Association SNP-/haplotype and endometrial cancer, chromosome 4. Associations between SNP-/haplotype and endometrial cancer, Chr 4 sorted on p-value. Due to the excel-sheet limitation of rows the complete data is not included.
(XLSX)

S5 Table Association SNP-/haplotype and endometrial cancer, chromosome 5. Associations between SNP-/haplotype and endometrial cancer, Chr 5 sorted on p-value. Due to the excel-sheet limitation of rows the complete data is not included.
(XLSX)

S6 Table Association SNP-/haplotype and endometrial cancer, chromosome 6. Associations between SNP-/haplotype and endometrial cancer, Chr 6 sorted on p-value. Due to the excel-sheet limitation of rows the complete data is not included.
(XLSX)

S7 Table Association SNP-/haplotype and endometrial cancer, chromosome 7. Associations between SNP-/haplotype and endometrial cancer, Chr 7 sorted on p-value. Due to the excel-sheet limitation of rows the complete data is not included.
(XLSX)

S8 Table Association SNP-/haplotype and endometrial cancer, chromosome 8. Associations between SNP-/haplotype and endometrial cancer, Chr 8 sorted on p-value. Due to the excel-sheet limitation of rows the complete data is not included.
(XLSX)

S9 Table Association SNP-/haplotype and endometrial cancer, chromosome 9. Associations between SNP-/haplotype and endometrial cancer, Chr 9 sorted on p-value. Due to the excel-sheet limitation of rows the complete data is not included.
(XLSX)

S10 Table Association SNP-/haplotype and endometrial cancer, chromosome 10. Associations between SNP-/haplotype and endometrial cancer, Chr 10 sorted on p-value. Due to the excel-sheet limitation of rows the complete data is not included.
(XLSX)

S11 Table Association SNP-/haplotype and endometrial cancer, chromosome 11. Associations between SNP-/haplotype and endometrial cancer, Chr 11 sorted on p-value. Due to the excel-sheet limitation of rows the complete data is not included.
(XLSX)

S12 Table Association SNP-/haplotype and endometrial cancer, chromosome 12. Associations between SNP-/haplotype and endometrial cancer, Chr 12 sorted on p-value. Due to the excel-sheet limitation of rows the complete data is not included.
(XLSX)

S13 Table Association SNP-/haplotype and endometrial cancer, chromosome 13. Associations between SNP-/haplotype and endometrial cancer, Chr 13 sorted on p-value. Due to the excel-sheet limitation of rows the complete data is not included.
(XLSX)

S14 Table Association SNP-/haplotype and endometrial cancer, chromosome 14. Associations between SNP-/haplotype and endometrial cancer, Chr 14 sorted on p-value. Due to the excel-sheet limitation of rows the complete data is not included.
(XLSX)

S15 Table Association SNP-/haplotype and endometrial cancer, chromosome 15. Associations between SNP-/haplotype and endometrial cancer, Chr 15 sorted on p-value. Due to the excel-sheet limitation of rows the complete data is not included.
(XLSX)

S16 Table Association SNP-/haplotype and endometrial cancer, chromosome 16. Associations between SNP-/haplotype and endometrial cancer, Chr 16 sorted on p-value. Due to the excel-sheet limitation of rows the complete data is not included.
(XLSX)

S17 Table Association SNP-/haplotype and endometrial cancer, chromosome 17. Associations between SNP-/haplotype and endometrial cancer, Chr 17 sorted on p-value. Due to the excel-sheet limitation of rows the complete data is not included.
(XLSX)

S18 Table Association SNP-/haplotype and endometrial cancer, chromosome 18. Associations between SNP-/haplotype and endometrial cancer, Chr 18 sorted on p-value. Due to the excel-sheet limitation of rows the complete data is not included.
(XLSX)

S19 Table Association SNP-/haplotype and endometrial cancer, chromosome 19. Associations between SNP-/haplotype and endometrial cancer, Chr 19 sorted on p-value. Due to the excel-sheet limitation of rows the complete data is not included.
(XLSX)

S20 Table Association SNP-/haplotype and endometrial cancer, chromosome 20. Associations between SNP-/haplotype and endometrial cancer, Chr 20 sorted on p-value. Due to the excel-sheet limitation of rows the complete data is not included.
(XLSX)

S21 Table Association SNP-/haplotype and endometrial cancer, chromosome 21. Associations between SNP-/haplotype and endometrial cancer, Chr 21 sorted on p-value. Due to the excel-sheet limitation of rows the complete data is not included.
(XLSX)

S22 Table Association SNP-/haplotype and endometrial cancer, chromosome 22. Associations between SNP-/haplotype and endometrial cancer, Chr 22 sorted on p-value. Due to the excel-sheet limitation of rows the complete data is not included.
(XLSX)

S23 Table Association SNP-/haplotype and endometrial cancer, chromosome 23. Associations between SNP-/haplotype and endometrial cancer, Chr 23 sorted on p-value. Due to the excel-sheet limitation of rows the complete data is not included.
(XLSX)

S24 Table Variants in haplotypes shown in Table 1. Each genetic locus is presented with variants included in the discovery haplotypes shown in Table 1. Rs-numbers are shown in the last column.
(DOCX)

S25 Table SNPs with lowest p-value within the risk loci demonstrated in Table 1. Each genetic locus is presented with gene in the area (if any), SNP, risk allele and corresponding frequency (F), odds ratio (OR) and p-value (P). Statistically significant SNP in 16q24.3 in bold.
(DOCX)

S26 Table Replication of suggested novel Endometrial cancer susceptibility loci (Sweden, Belgium and Germany). Replication of suggested novel Endometrial cancer susceptibility loci with a sliding window haplotype approach (window size 1-25) at 2q31.1, 4p16.1, 4p15.31, 6q13, 7p21.1, 9p13.3, 10q26.3, 11q21, 12q13.11, 13q12.11, 15q13.3, 16q24.3, 19q13.32, 20p12.3 and 22q13.2 in a Swedish, a Belgian and a German cohort.
(XLSX)

S27 Table Published ECAC SNPs in comparison with Swedish data from present study. Each locus presented with gene in the area, corresponding effect allele frequency (EAF), odds ratio (OR), and p-value (P) Reference panel GRCh37 for position (BP).
(DOCX)
