# Supplementary material for: A Swedish genome-wide haplotype association analysis identifies novel candidate loci associated with endometrial cancer risk
Source: PLoS One. 2025 Mar 18;20(3):e0316086. doi: 10.1371/journal.pone.0316086 (PMC13537468; doi:10.1371/journal.pone.0316086)
Supplement: Supplementary file 1 [file pone.0316086.s001.pdf]

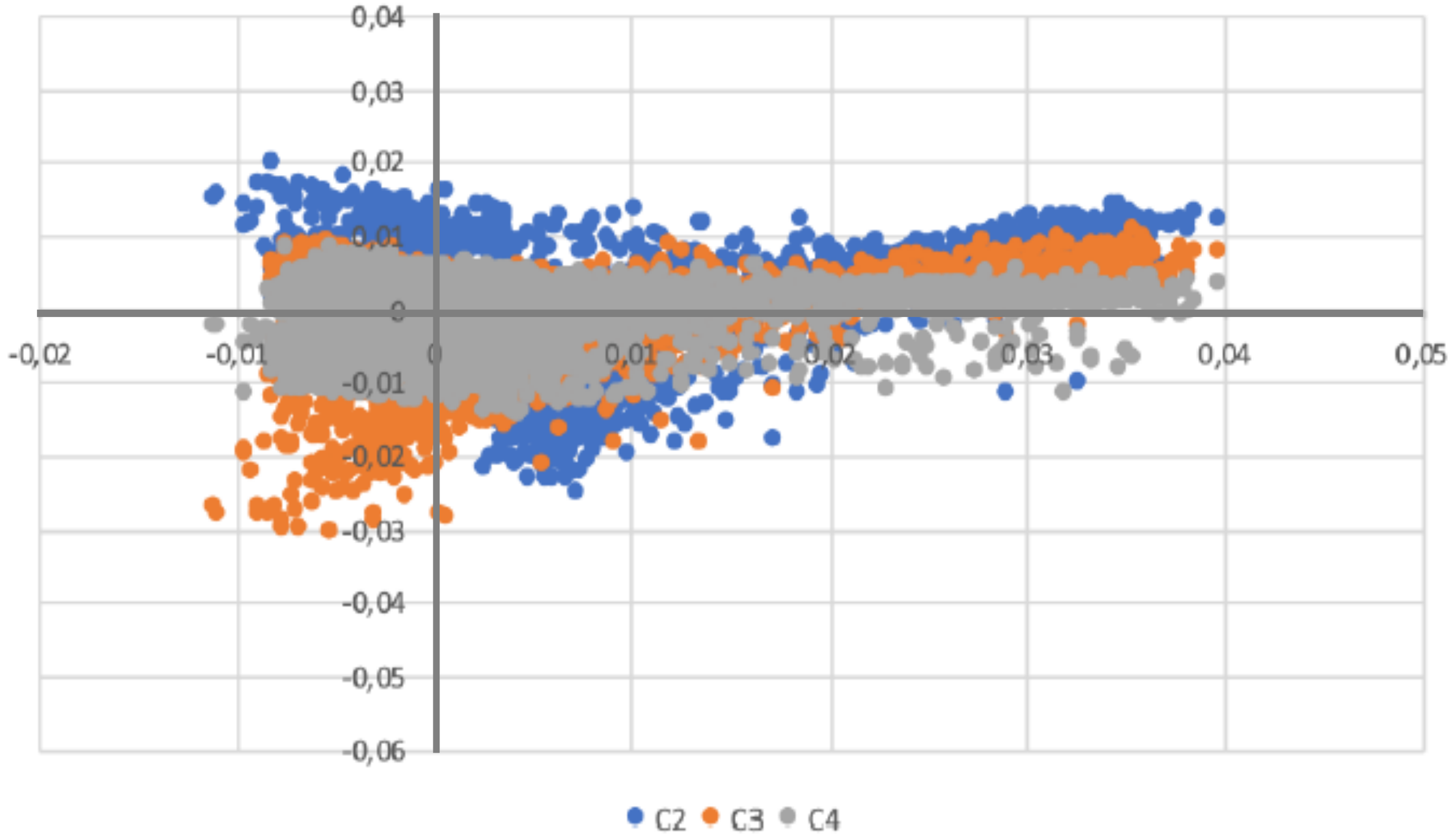
0,04
0,03
0,02
0,01
0
-0,01
-0,02
-0,03
-0,04
-0,05
-0,06
-0,02
-0,01
0
0,01
0,02
0,03
0,04
0,05
C2
C3
C4
