# Supplementary material for: A Swedish genome-wide haplotype association analysis identifies novel candidate loci associated with endometrial cancer risk
Source: PLoS One. 2025 Mar 18;20(3):e0316086. doi: 10.1371/journal.pone.0316086 (PMC13537468; doi:10.1371/journal.pone.0316086)
Supplement: Supplementary file 2 [file pone.0316086.s002.pdf]

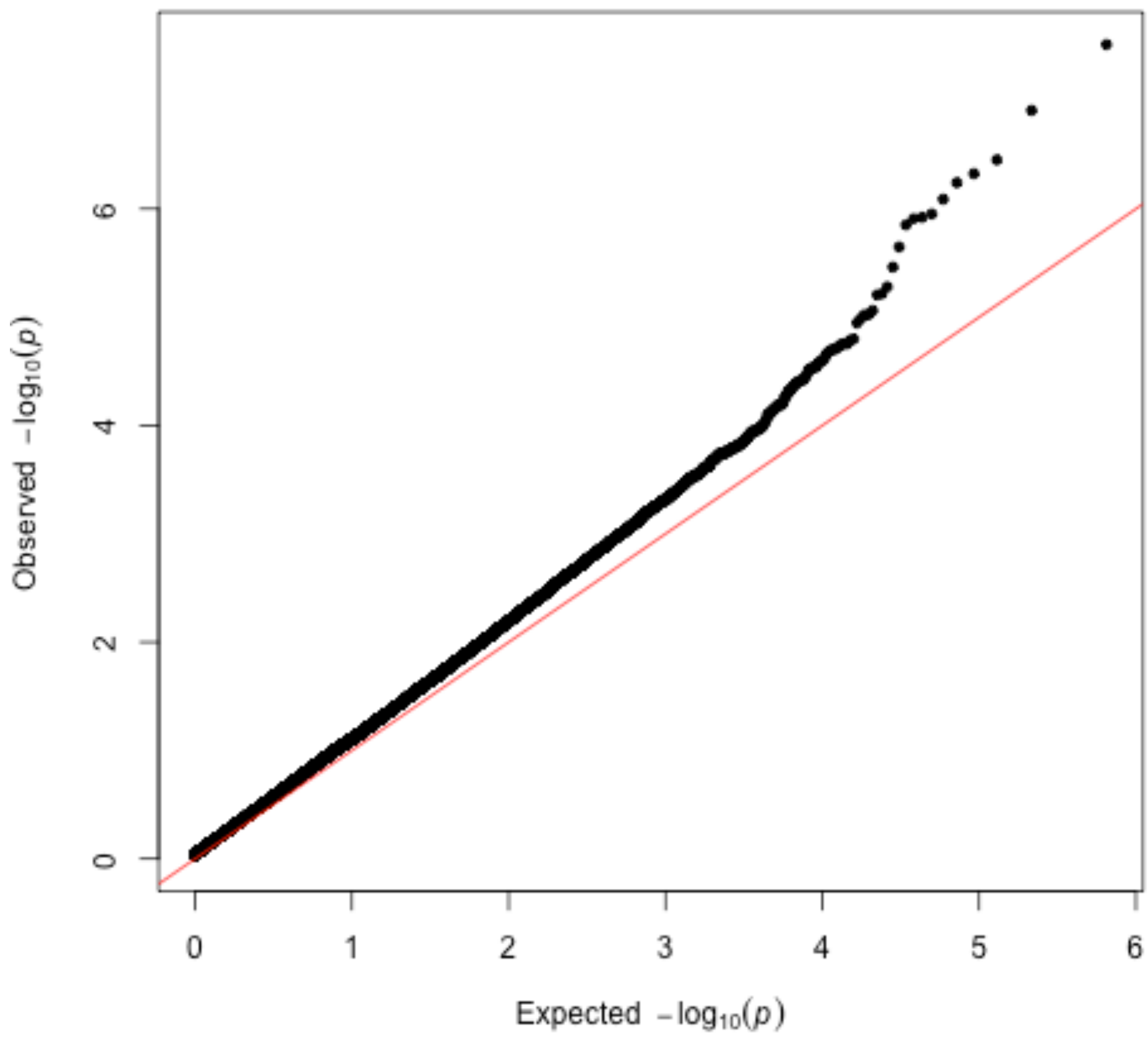

Observed $-\log_{10}(p)$
0
2
4
6
Expected $-\log_{10}(p)$
0
1
2
3
4
5
6
