# Supplementary material for: A Swedish genome-wide haplotype association analysis identifies novel candidate loci associated with endometrial cancer risk
Source: PLoS One. 2025 Mar 18;20(3):e0316086. doi: 10.1371/journal.pone.0316086 (PMC13537468; doi:10.1371/journal.pone.0316086)
Supplement: Supplementary file 3 [file pone.0316086.s003.pdf]

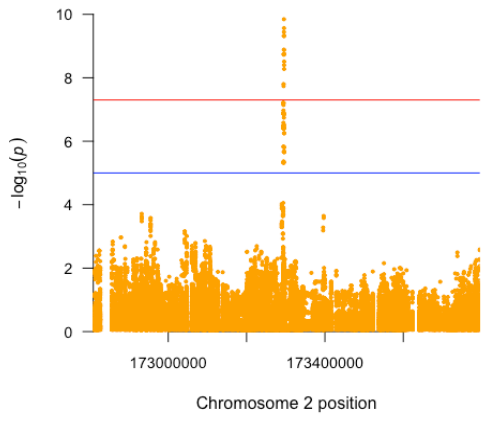

(A) Locus 2q31.1

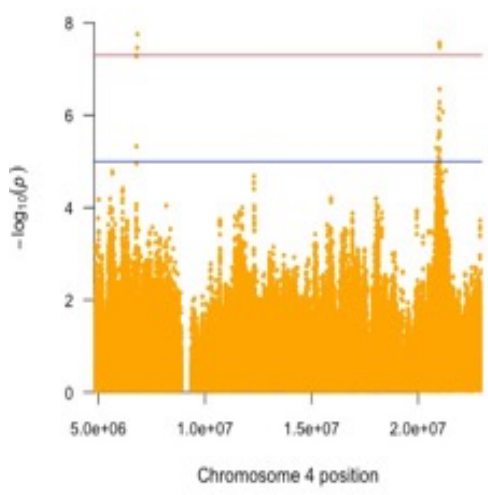

(B) Locus 4p16.1 & 4p15.31

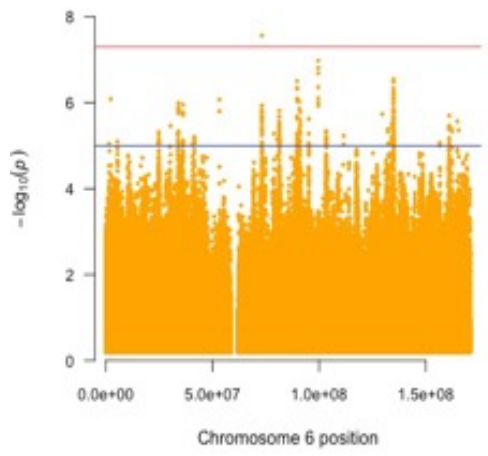

(C) Locus 6q13

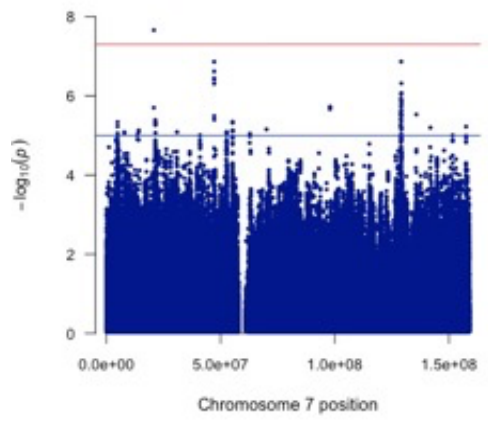

(D) Locus 7p21.1

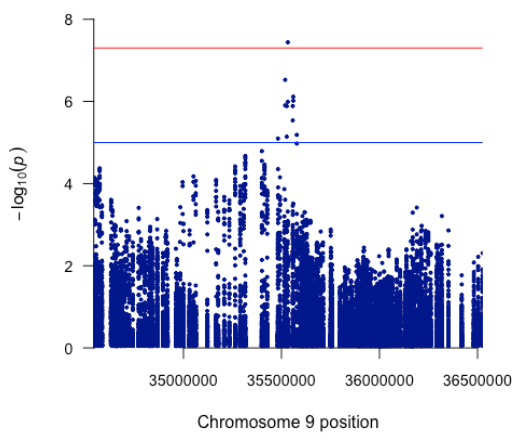

(E) Locus 9p13.3

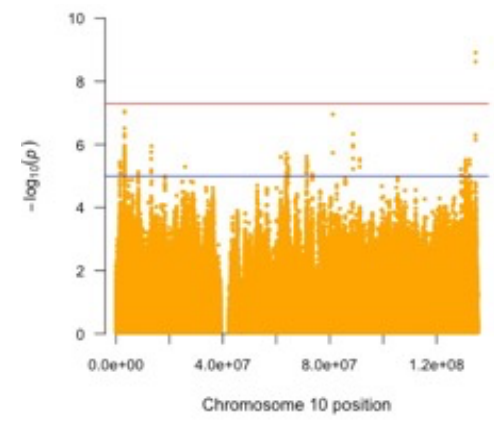

(F) Locus 10q26.3

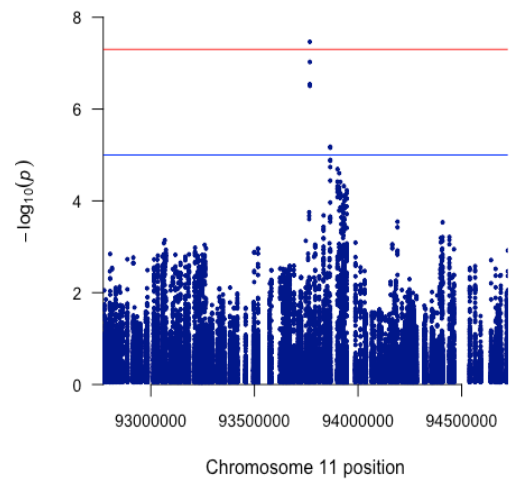

(G) Locus 11q21

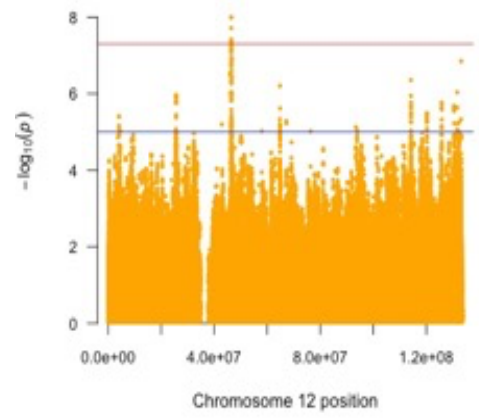

(H) Locus 12q13.11

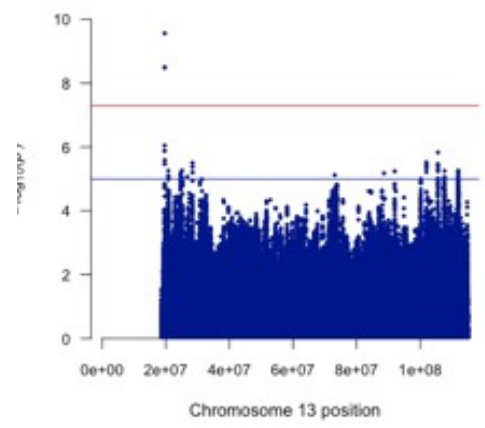

(I) Locus 13q12.11

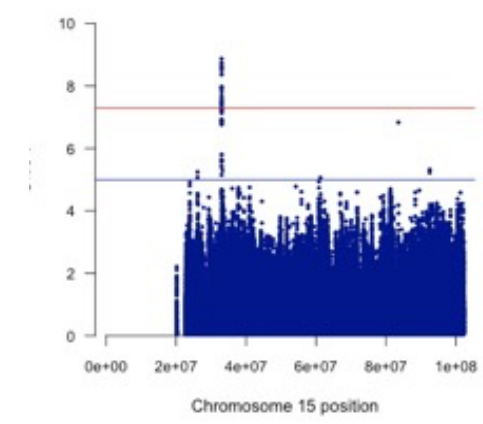

(J) Locus 15q13.3

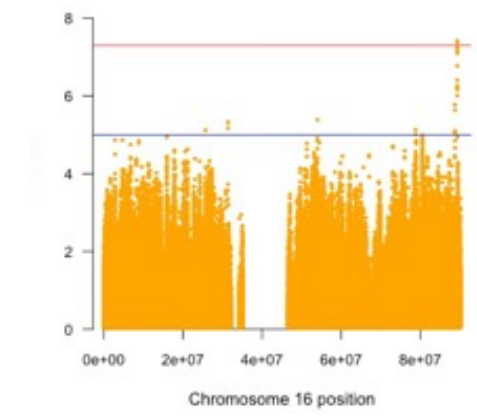

(K) Locus 16q24.3

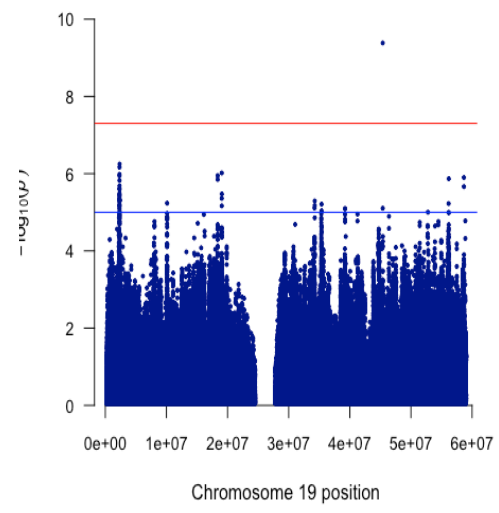

(L) Locus 19q13.32

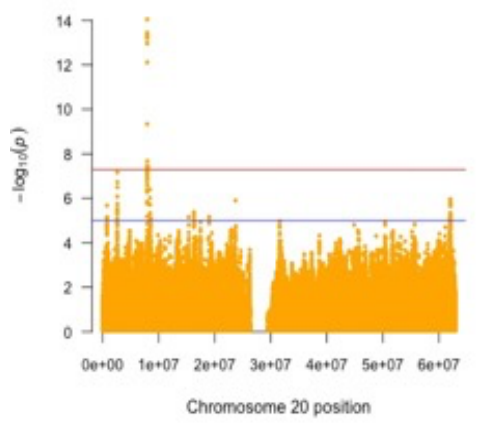

(M) Locus 20q12.3

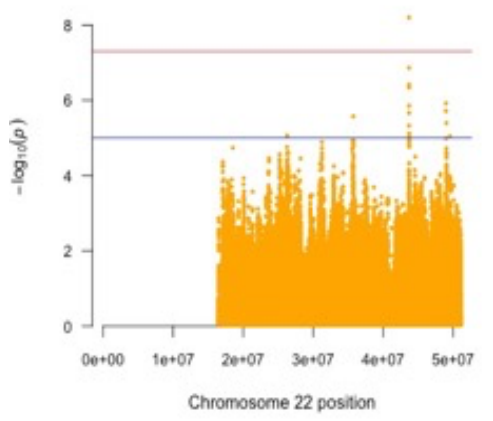

(N) Locus 22q13.2
