# Supplementary material for: A Swedish genome-wide haplotype association analysis identifies novel candidate loci associated with endometrial cancer risk
Source: PLoS One. 2025 Mar 18;20(3):e0316086. doi: 10.1371/journal.pone.0316086 (PMC13537468; doi:10.1371/journal.pone.0316086)
Supplement: Supplementary file 4 [file pone.0316086.s004.pdf]

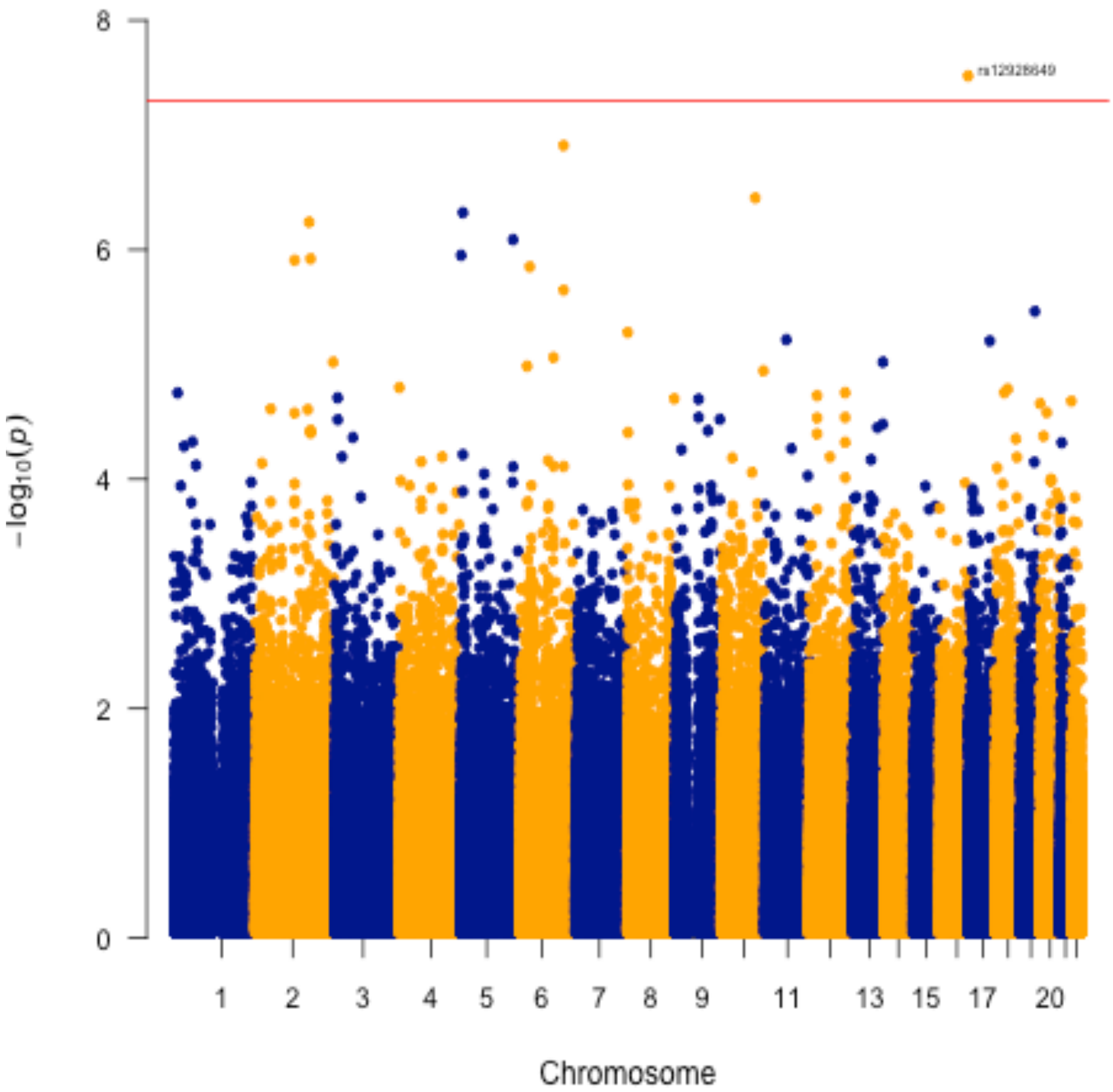
rs12928649
8
6
4
2
0
$-\log_{10}(p)$
1
2
3
4
5
6
7
8
9
11
13
15
17
20
Chromosome
