# Supplementary material for: A Swedish genome-wide haplotype association analysis identifies novel candidate loci associated with endometrial cancer risk
Source: PLoS One. 2025 Mar 18;20(3):e0316086. doi: 10.1371/journal.pone.0316086 (PMC13537468; doi:10.1371/journal.pone.0316086)
Supplement: Supplementary file 5 [file pone.0316086.s028.docx]

| Locus | Haplotype,discovery | variants in haplotype,  discovery GWAS | Variants analyzed in replication analysis in discovery haplotype region | rs number |
| --- | --- | --- | --- | --- |
| 2q31.1 | G  A  A  G  G  T  G | rs12053442  rs3108780  rs6757785  rs76264695  rs35265291  rs3115745  rs36055280 | rs12053442  rs3108780  rs6757785  rs76264695  rs35265291  rs3115745  rs36055280 |  |
| 4p16.1 | G  G  -  G  A  G  G  G | rs6833118  chr4_6834367_C_T  -  rs11723013  rs6845985  rs10022837  rs13115900  rs7675928 | rs6833118  chr4_6834367_C_T  chr4_6836005_C_T  rs11723013  rs6845985  rs10022837  rs13115900  rs7675928 | rs62289446  rs115390572 |
| 4p15.31 | A  C  G  G  A  A  G  G  A  A  G  A  T  C  A  -  G  G  G  C  G  -  A  C  G | rs10938823  rs11938766  chr4_21016367_C_T  rs4348088  rs12233886  rs10033946  rs12510687  rs6817687  rs720853  rs7696887  rs16870421  rs7687191  rs11945930  rs6448023  rs10011670  -  rs17506318  rs876478  rs7667000  rs1347240  rs2113966  -  rs10013055  rs11724880  rs1156764 | rs10938823  rs11938766  chr4_21016367_C_T  rs4348088  rs12233886  rs10033946  rs12510687  rs6817687  rs720853  rs7696887  rs16870421  rs7687191  rs11945930  rs6448023  rs10011670  rs10007205  rs17506318  rs876478  rs7667000  rs1347240  rs2113966  rs10021586  rs10013055  rs11724880  rs1156764 | rs76349941 |
| 6q13 | A  C  A  G  A  A  A  -  A  A  C  G  G  G  G  G | rs1572208  rs2815715  rs2815717  rs7760670  rs9360565  rs9350463  rs9446692  -  rs1202116  rs12205054  chr6_73300665_G_T  rs1202100  rs10943040  chr6_73325585_C_T  rs7772714  rs6913237 | rs1572208  rs2815715  rs2815717  rs7760670  rs9360565  rs9350463  rs9446692  chr6_73234147_A_G  rs1202116  rs12205054  chr6_73300665_G_T  rs1202100  rs10943040  chr6_73325585_C_T  rs7772714  rs6913237 | rs1328854  rs1742290  rs12524568 |
| 7p21.1 | A  A  G  G  G  G  G | kgp129516  chr7_20801627_A_G  chr7_20807298_A_G  rs2709748  chr7_20820129_C_T  rs2286260  chr7_20824614_C_T | kgp129516  chr7_20801627_A_G  chr7_20807298_A_G  rs2709748  chr7_20820129_C_T  rs2286260  chr7_20823258_C_T  chr7_20824614_C_T | rs966717  rs62453402  rs2528798  rs6944569  rs143163563  rs34908430 |
| 9p13.3 | G  A  A  G  C  A  G  G  G  G  A  A  G  A  A | rs10972503  rs1890590  rs12338773  rs10758312  rs7849006  rs10814259  rs2275422  rs4879913  rs10814260  rs10972530  rs10972531  rs12551214  rs2153240  rs3829076  chr9_35658163_A_T | rs10972503  corect_rs117345731  rs1890590  rs12338773  rs10758312  rs7849006  rs10814259  rs2275422  rs4879913  rs10814260  rs10972530  rs10972531  rs12551214  rs2153240  rs3829076  chr9_35658163_A_T | rs117345731  rs3750430 |
| 10q26.3 | A  G  A  G  A  A  A | rs913191  rs2767438  rs2254419  rs4880433  rs10870341  rs7095353  rs7907613 | rs913191  rs2767438  rs2254419  rs4880433  rs7095353  rs7914850  rs7907613 |  |
| 11q21 | G  G  G  C  A  C  G  A  C  G  G  G  G  A  C  A  A  A  G  G  A  A  A  C | rs4753116  rs7127348  rs2460042  rs1401186  rs10765653  rs12222234  rs4753538  rs1138800  rs4073612  rs7482084  rs882937  rs1046812  rs3020004  rs1792632  rs1792634  rs1857682  rs1014499  rs4753133  rs6483320  rs1361929  rs519834  rs7930551  rs495862  rs484889 | rs4753116  rs7127348  rs2460042  rs1401186  rs10765653  rs12222234  rs4753538  rs1138800  rs4073612  rs7482084  rs882937  rs1046812  rs3020004  rs1792632  rs1792634  rs1857682  chr11_93933708_A_G  rs1014499  rs4753133  chr11_93958601_A_G  rs11826531  rs6483320  rs1361929  rs519834  rs1939393  rs7930551  rs495862  rs484889 | rs117459535  rs186045601 |
| 12q13.11 | G  A  G  A  G  G  A  A  C  C  A  G  G  G  C | rs7967938  rs1444588  chr12_46464980_A_G  rs10880903  rs7311811  rs1373549  chr12_46506005_A_G  chr12_46514123_A_G  rs2408468  rs10880923  rs10785611  rs10128931  rs7958795  rs2197399  rs2166138 | rs7967938  rs1444588  chr12_46464980_A_G  rs10880903  rs7311811  rs1373549  rs1822738  chr12_46506005_A_G  chr12_46514123_A_G  rs2408468  rs10880923  rs10785611  rs10128931  rs7958795  rs2197399  rs2166138 | rs7313125  rs11183345  rs2408466 |
| 13q12.11 | A  G  G  A  G  A  G  A  G  A  A  G  A | rs17090505  rs1974048  rs7320243  rs9319225  rs4770842  chr13_19727056_A_G  rs518637  rs9511884  rs2006655  chr13_19748709_G_T  rs9507572  rs491873  rs7338881 | rs17090505  chr13_19694953_A_T  chr13_19699241_C_G  rs1974048  rs7320243  rs9319225  rs4770842  chr13_19724838_C_T chr13_19727056_A_G  rs518637  rs479287  rs9511884  rs2006655  chr13_19748709_G_T  rs9507572  rs491873  rs7338881 | rs9507474  rs74037709  rs74037721  rs61742516  rs2774494 |
| 15q13.3 | G  A  A  A  A  G  A  A  G  A  A  A  A  A  A  A  G  G  T  G  G  A  C | rs55659128  rs28650777  rs12906413  rs3861195  rs12902616  rs11853552  rs11857190  rs11857997  rs8031842  rs1554865  rs1881538  rs12914734  rs11638007  rs11632715  rs1534594  rs12594148  rs12592288  rs3812934  rs1406389  15_33010736_G_A  rs7168877  rs9806137  rs8034965 | rs55659128  rs28650777  rs12906413  rs3861195  rs12902616  rs11853552  rs11857190  rs11857997  rs8031842  rs8031842  rs1554865  rs1881538  rs12914734  rs11638007  rs11632715  rs1534594  rs12594148  rs12592288  rs3812934  rs72715291  rs1406389  corect_rs2293581(=15_33010736_G_A)  rs7168877  rs9806137  corect_chr15:33011740:D | rs2293581  rs72360084 |
| 16q24.3 | G | rs12928649 | rs4785648  rs7192878 |  |
| 19q13.32 | A  A  A  G  A  G  A | chr19_45382984_G_T  chr19_45384405_C_T  chr19_45395619_A_G  rs157582  rs1160985  chr19_45410002_A_G  rs7412 | chr19_45382984_G_T  chr19_45384405_C_T  chr19_45395619_A_G  rs157582  rs1160985  chr19_45410002_A_G | rs11669338  rs73052307  rs2075650  rs769449 |
| 20p12.3 | A  G  A  A  A  G  C  G  G  C  G  A  C | rs4813847  rs1012891  rs6055457  rs6039000  rs2205782  rs67898032  rs6133527  rs2294248  rs2205783  rs6039005  rs6055475  rs6140508  rs6055481 | rs4813847  rs17334672  rs1012891  rs6055457  rs7266941  rs6039000  rs2205782  rs67898032  rs6133527  rs2294248  rs80032517  rs2205783  rs6039004  rs6039005  rs6055475  rs6140508  rs6055481 |  |
| 22q13.2 | G  G  G  C  G  G  C  G  A  A  A  G  A  A  A  A  G  A  A  G  G | chr22_43689984_C_T  chr22_43693733_A_G  chr22_43698129_C_T  chr22_43699006_C_G  rs6003147  rs11704418  chr22_43713253_C_G  chr22_43715848_A_G  chr22_43715862_A_G  rs3213584  chr22_43717197_C_T  chr22_43720447_C_G  rs6003156  chr22_43722625_A_G  rs9612042  rs6003160  rs5759291  rs715001  rs4820523  rs5759305  rs695648 | chr22_43689984_C_T  chr22_43692585_G_T  chr22_43693733_A_G  chr22_43698129_C_T  chr22_43698662_C_G  chr22_43699006_C_G  rs2269670  rs6003147  rs11704418  chr22_43713253_C_G  chr22_43714693_A_C  chr22_43715848_A_G  chr22_43715862_A_G  chr22_43716273_C_G  rs3213584  chr22_43717197_C_T  chr22_43720447_C_G  rs6003156  chr22_43722625_A_G  rs9612042  rs6003160  chr22_43733940_AGACCTGGATTTC_INDEL  rs5759291  chr22_43738701_C_T  chr22_43740441_INDEL_TT  chr22_43742510_A_G  rs715001  chr22_43746418_A_G  rs4820523  chr22_43747981_A_INDEL  chr22_43754675_A_G  rs5759305  rs695648 | rs5996306  rs2007836  rs2253884  rs5759271  rs74476661  rs5759271  rs2744875  rs2859441  rs2744874  rs2744875  rs5759281  rs9623809  rs2744874  rs5759284  rs5759281  rs9623809  rs5759284  rs11268193  rs150921780  rs113046994  rs7284129  rs117337715  rs200221371  rs73166132 |
