# Supplementary material for: A Swedish genome-wide haplotype association analysis identifies novel candidate loci associated with endometrial cancer risk
Source: PLoS One. 2025 Mar 18;20(3):e0316086. doi: 10.1371/journal.pone.0316086 (PMC13537468; doi:10.1371/journal.pone.0316086)
Supplement: Supplementary file 6 [file pone.0316086.s029.docx]

| Locus | Gene | SNP | Allele | F | OR | P-value |
| --- | --- | --- | --- | --- | --- | --- |
| 2q31.1 | *ITGA6* | rs36055280 | G | 0.66 | 1.05 | 0.145 |
| 4p16.1 | *KIAA0232* | rs62289446 | G | 0.95 | 1.19 | 0.13 |
| 4p15.31 | *KCNIP4* | rs12510687 | G | 0.26 | 1.19 | 0.00144 |
| 6q13 | *KCNQ5* | rs1202116 | A | 0.55 | 1.12 | 0.0175 |
| 7p21.1 | *ABCB5* | rs34908430 | G | 0.68 | 1.19 | 0.00189 |
| 9p13.3 | *RUSC2, FAM166B, CD72,*  *TESK1, SIT1, RMRP, CCDC107* | rs10758312 | G | 0.31 | 1.16 | 0.0044 |
| 10q26.3 | *TTC40* | rs10870341 | G | 0.12 | 1.22 | 0.00969 |
| 11q21 | *HEPHL1, PANX1 FOLR4* | rs7482084 | G | 0.68 | 1.13 | 0.19 |
| 12q13.11 | *-* | rs7958795 | G | 0.59 | 1.18 | 8.39x10^-4^ |
| 13q12.11 | *TUBA3C* | rs2006655 | G | 0.82 | 1.2 | 0.00629 |
| 15q13.3 | *GREM1* | rs1528734 | A | 0.32 | 1.09 | 0.0804 |
| 16q24.3 | ***ANKRD11*** | **rs12928649** | **G** | **0.083** | **1.58** | **3.36x10^-8^** |
| 19q13.32 | *PVRL2, TOMM40, APOE* | rs7412 | A | 0.08 | 1.47 | 3.8x10^-6^ |
| 20p12.3 | *TMX4* | rs16994371 | G | 0.67 | 1.14 | 0.0126 |
| 22q13.2 | *SCUBE1* | rs2253884 | G | 0.84 | 1.19 | 0.0099 |
