# Supplementary material for: A Swedish genome-wide haplotype association analysis identifies novel candidate loci associated with endometrial cancer risk
Source: PLoS One. 2025 Mar 18;20(3):e0316086. doi: 10.1371/journal.pone.0316086 (PMC13537468; doi:10.1371/journal.pone.0316086)
Supplement: Supplementary file 7 [file pone.0316086.s031.docx]

| Locus | SNP | BP | Nearby gene | Effect:other allel | EAF | | OR | | P | | Ref |
| --- | --- | --- | --- | --- | --- | --- | --- | --- | --- | --- | --- |
|  |  |  |  |  | ECAC | SWE | ECAC | SWE | ECAC | SWE |  |
| 1p34.3 | rs113998067 | 38073356 | *GNL2*, *RSPO1*, *CDCA8* | C:T | 0.04 | - | 1.23 | - | 3.6x10^-8^ | - | (1) |
| 2p16.1 | rs148261157 | 60897579 | *BCL11A* | A:G | 0.03 | - | 1.26 | - | 3.4x10^-8^ | - | (1) |
| 6q22.3 | rs1740828 | 21649085 | *SOX4* | G:A | 0.52 | 0.508 | 1.15 | 1.17 | 4.2x10^-16^ | 1.06x10^-3^ | (1) |
| 6q22.31 | rs2747716 | 126008372 | *HEY2, NCOA7* | A:G | 0.57 | - | 1.10 | - | 2.9x10^-10^ | - | (1) |
| 6q22.31 | rs13328298 | 126016580 | *HEY2, NCOA7* | G:A | 0.58 | - | 1.13 | - | 3.73x10^-10^ | - | (2) |
| 8q24.21 | rs35286446 | 129445863 | *MYC* | GAT:G | 0.58 | - | 1.10 | - | 3.1x10^-9^ | - | (1) |
| 8q24.21 | rs4733613 | 129599278 | *MYC* | C:G | 0.12 | 0.865 | 1.18 | 0.878 | 7.5x10^-14^ | 0.0541 | (1) |
| 8q24.21 | rs139584729 | 129623902 | *MYC* | C:G | 0.98 | - | 1.40 | - | 2.4x10^-8^ | - | (1) |
| 9p21.3 | rs1679014 | 22207037 | *CDKN2A*, *CDKN2B* | T:C | 0.07 | 0.0725 | 1.18 | 1.3 | 6.4x10^-9^ | 2.44x10^-3^ | (1) |
| 11p13 | rs10835920 | 32489664 | *WT1*, *WT1-AS*, *RCN1*, *CCDC73*, *EIF3M*, *TCP11L1* | T:C | 0.38 | - | 1.09 | - | 1.3x10^-8^ | - | (1) |
| 12p12.1 | rs9668337 | 26426338 | *SSPN* | A:G | 0.74 | - | 1.11 | - | 1.1x10^-9^ | - | (1) |
| 12q24.11 | rs3184504 | 111884608 | *SH2B3* | C:T | 0.52 | 0.532 | 1.10 | 1.18 | 1.1x10^-10^ | 7.06x10^-4^ | (1) |
| 12q24.21 | rs10850382 | 115214548 | *LOC107984437* | T:C | 0.31 | 0.307 | 1.10 | 1.11 | 3.5x10^-9^ | 0.0436 | (1) |
| 13q22.1 | rs7981863 | 73812141 | *KLF5, KLF12* | C:T | 0.72 | 0.771 | 1.16 | 1.25 | 2.7x10^-17^ | 2.1x10^-4^ | (1) |
| 13q22.1 | (rs11841589) | 73814891 | *KLF5, KLF12* | G:T | 0.75 | 0.78 | 1.15 | 1.24 | 4.83x10^-11^ | 3.28x10^-4^ | (2) |
| 15q15.1 | rs937213 | 40322124 | *EIF2AK4, BMF* | C:T | 0.42 | 0.421 | 1.09 | 1.03 | 5.1X10^-9^ | 0.494 | (1) |
| 15q21 | rs2414098 | 51537806 | *CYP19A1* | C:T | 0.62 | - | 1.17 | - | 4.51x10^-13^ | - | (3) |
| 15q21.2 | rs17601876 | 51553909 | *CYP19A1* | G:A | 0.48 | - | 1.12 | - | 3.3X10^-14^ | - | (1) |
| 17q11.2 | rs1129506 | 29646032 | *EVI2A*, *NF1*, *SUZ12*, *RP11-848P1.5* | G:A | 0.38 |  | 1.10 |  | 4.3x10^-8^ |  | (1) |
| 17q12 | rs11263761 | 36097775 | *HNF1B* | A:G | 0.52 | - | 1.15 | - | 3.4x10^-14^ | - | (1) |
| 17q12 | rs11263763 | 36103565 | *HNF1B* | A:G | 0.54 | 0.56 | 1.20 | 1.08 | 2.78x10^-19^ | 0.0961 | (4) |
| 17q21.32 | rs882380 | 46294236 | *SKAP1*, *SNX1* | A:C | 0.61 | - | 1.10 | - | 4.7x10^-9^ | - | (1) |

**References**

1.	O'Mara TA, Glubb DM, Amant F, Annibali D, Ashton K, Attia J*, et al.* Identification of nine new susceptibility loci for endometrial cancer. Nat Commun **2018**;9:3166

2.	Cheng TH, Thompson DJ, O'Mara TA, Painter JN, Glubb DM, Flach S*, et al.* Five endometrial cancer risk loci identified through genome-wide association analysis. Nat Genet **2016**;48:667-74

3.	Thompson DJ, O'Mara TA, Glubb DM, Painter JN, Cheng T, Folkerd E*, et al.* CYP19A1 fine-mapping and Mendelian randomization: estradiol is causal for endometrial cancer. Endocr Relat Cancer **2016**;23:77-91

4.	Painter JN, O'Mara TA, Batra J, Cheng T, Lose FA, Dennis J*, et al.* Fine-mapping of the HNF1B multicancer locus identifies candidate variants that mediate endometrial cancer risk. Hum Mol Genet **2015**;24:1478-92
