# Supplementary material for: A Swedish genome-wide haplotype association analysis identifies novel candidate loci associated with endometrial cancer risk
Source: PLoS One. 2025 Mar 18;20(3):e0316086. doi: 10.1371/journal.pone.0316086 (PMC13537468; doi:10.1371/journal.pone.0316086)
Supplement: Supplementary file 8 [file pone.0316086.s032.docx]

Response to Reviewer 2 Comments

Thank you for your review of our paper and your valuable comments. Your comments indicate that we have not accurately described our intent and study design. We have improved the manuscript, see below points:

**Reviewers comment 1:** In this manuscript by Barnekow et al., the authors performed a GWAS haplotype analysis in three different populations: Swedish, Belgian, and German. From the haplotype analysis, they identified 15 novel loci which they claimed are associated with endometrial cancer (EC) risk.

**Response 1:** There is a two-stage GWAS performed with a Swedish discovery set and two replication sets for validation - a German and a Belgian. We have clarified the study design throughout the manuscript (Abstract, Introduction, Results, Discussion).

**Reviewers comment 2:** While the methods and bioinformatics analysis in this study were well-conducted, the outcomes lack significance and the identified loci or haplotypes do not seem to correspond to EC and could potentially be misleading.

**Response 2:** We disagree. The findings are new and therfore of interest. The 15 Swedish novel endometrial cancer susceptibility loci were replicated with strong suupport in 8 in of the 15 loci (and in the remaining 7 with lower support). We therefore reject the null-hypothesis that no association occurs.

**Reviewers comment 3:** The authors did not differentiate between control and patient samples for the haplotype analysis.

**Response 3:** Cases and controls are differentiated. This endometrial cancer GWAS is a case-control study and the standard procedures for a GWAS analysis is used. The samples are merged but the individuals are defined as endometrial cancer cases (1) or controls (0), respectively. Thereafter, the odds ratios are calculated in PLINK based on the haplotype frequency in cases vs controls. Please see section Material and Methods for detailed information.

**Reviewers comment 4:** The 15 loci identified are from mixed populations, which could lead to a mischaracterization of EC risk.

**Response 4:** We agree that haplotype analysis requires homogenous populations. The 15 loci are identified in the Swedish population. For validation of the novel Swedish loci replication was performed in two genetically similar populations. We added the text (in red) to introduction to clarify, page 3, line 77-80:

“For validation, a replication GWAS of the Swedish novel loci were performed in two small European cohorts. The replication cohorts were selected from genetically similar populations (Belgian and German) from the ECAC study to enable haplotype comparison and to identify potential founder mutations [5].”

**Reviewers comment 5:** Surprisingly, none of the 15 loci (Table 1) identified a candidate oncogene or tumor suppressor gene specifically involved in EC! Instead, all these genes are known to be misregulated in other cancers such as skin, breast, colon, and hepatocellular carcinoma as discussed by the authors. This indicates a potential overlap in gene dysregulation across various cancer types but is not specific to EC.

**Response 5:** We agree, the identified novel suscpeptibility loci could be shared with other tumour types. Nevertheless, this is the first performed endometrial cancer haplotype GWAS and the study objective is to identify suseptibility loci associated with endometrial cancer, which we found. Most important the replication analysis found strong support in 8 of the 15 loci (and lower support in 7) and many of the regions is previously reported in the realtion to cancer, which we believe support the findings. To determine whether any of the loci share genetic risk loci with other tumour types as a part of a cancer syndrome further studies are warranted.
