# Supplementary material for: A Swedish genome-wide haplotype association analysis identifies novel candidate loci associated with endometrial cancer risk
Source: PLoS One. 2025 Mar 18;20(3):e0316086. doi: 10.1371/journal.pone.0316086 (PMC13537468; doi:10.1371/journal.pone.0316086)
Supplement: Supplementary file 9 [file pone.0316086.s033.docx]

Response to Editor

Thank you for your very thorough review of our paper and your valuable comments.

**Editor´s Comments**
The manuscript is interesting and tackles a very important disorder. The haplotype focus is also an advanced topic of broader relevance. The reviewers were quite enthusiastic, but raised a few points that need clarifying mainly. No need for more analyses or additional work beyond adjustments to text and visuals.

The major points are:

**Editor´s comment 1**

We recommend providing a summary of the discovery analysis, such as the number of windows and haplotypes being tested (rev2)

**Response 1**

We agree, please see response to reviewer 2 (response 1).

**Editor´s comment 2**

Clarify how corrections for multiple testing were handled in replication cohorts (rev1), see also concern from rev3 about “overlapping/repeated” signals. Also, are the p values and OR´s in figure 1 from pairwise tests of haplotypes or what??

**Response 2**

Please see response to reviewer 1 (response 2) and response to reviewer 3 (response 1).

**Editor´s comment 3**

Rev 3 raised a concern about the replication cohort. Please clarify.

**Response 3**

Please see response to reviewer 3 (response 2).

**Editor´s comment 4**

Figure 1 legend should include the sample sizes for each of the cohorts (rev1)

**Response 4**

We agree, please see response to reviewer 1 (response 1b).

**Editor´s comment 5**

It is worthwhile to discuss also the potential reasons for haplotype associations. Is it due to the genetic influence from multiple single markers or combinatorial effects of many markers? The reviewers add several good pointers and suggestions. Note, on comment 3 from rev 2. We suggest adding genomic coordinates (but not dropping the SNP ids, they are helpful).

**Response 4**

a) We agree, we added the text to introduction, page 3, line 78-80:

“Thus, an identified unique risk haplotype defines the borders of a DNA region holding an assumed risk variant between the first and last marker, most probably not any of the included markers.”

b) Thanks, the genomic positition is added. Please see response to reviewer 2 (response 3b).

Response to Reviewer 1

Thank you for your very thorough review of our paper and your valuable comments.

Originality: It is perhaps obvious that haplotypes contain more information than individual SNPs for GWAS studies (as the authors and other has shown in prior studies)

Scientific Quality: Careful attention to details, adequate sample sizes, important inclusion of a relevant replication group

General Comment: The paper is well written

**Reviewer´s comment 1:**
The manuscript list three replication cohorts Swedish, Belgian and German. It is not clear what samples are part of the Swedish replication cohort. Figure 1 as well lists 3 datasets as replication cohorts. The legend should include the sample sizes for each of the cohorts.

**Response 1:**

a) We added and moved text to Material and Methods to clarify, page 6, line 159-161:

“The same Swedish cohort from the discovery analysis was used even in the replication stage, but with slightly more cases and controls included (1,139 cases and 6,080 controls).”

b) We added the following text (in red) to the Figure 1 legend to clarify, page 9, line 250-251:

“This is Fig 1 legend. Replication GWAS in a German (221 cases ; 251 controls) and a Belgian cohort (528 cases ; 1,266 controls) along with the Swedish cohort (1,139 cases; 6,080 controls).”

**Reviewer´s comment 2:**
The authors suggest a p<0.05 as significance threshold for the replication cohorts. However, for each discovered haplotypes of interest the study tests multiple distinct haplotypes in the replication cohorts. In such scenario there should be multiple testing correction performed.

**Response 2:**

We agree, the significant threshold can be discussed. We have therefore modified the text with focus on possible founder regions with identical haplotypes and positive ORs.

We added the text (in red) to abstract, page 2, line 47-48:

“In spite of small sample sizes in the replication cohorts, there was still support for most loci with positive ORs.”

We added the text to material and methods, page 7, line 190-193:

“Because of the limited size of the Belgian and German cohorts, we did not expect statistically significant values generally used for GWAS in replication. Instead, we focused on the odds ratios and present the p-values from the PLINK analysis without correction for multiple testing across the 15 loci.”

We added the following text (in red) to discussion, page 10, line 275-285:

“Two European cohorts were available for validation analysis. Both were however small, which limited the ability to achieve statistical significance in this analysis. Still, the finding of haplotypes with positive ORs as in the discovery analysis within the tested regions suggested risk haplotypes at the same loci, and even suggested the possibility of European founders. Identification of potential founder mutations required a new QC including all the three cohorts to involve identical SNPs for comparison. This means that the Swedish replication result was not identical to the discovery result. This was expected, as GWAS is highly dependent on the markers used in the analysis. Importantly, all loci had positive ORs in the replication for all three cohorts. All loci which showed compelling associations in the Swedish discovery cohort did not reach the same level of statistical significance in the replication sets.”

We also added the following text to conclusion, page 15, line 427-429:

“The finding of possible European founder regions is of special interest and future sequencing of familial EC could identify the risk variants at these loci.”

**Reviewer´s comment 3:**
Only 4p15.31 and 12q13.11 loci share a common haplotype background in the replication cohorts as per figure 1. Possibly imputation should be performed for these regions to identify risk marker.

Response 3:
We agree, loci 4p15:31 and 12q13.11 are of interest for furher studies. Imputation in this unselected cohort could further define the region. However, to find the actual risk causing variant we suggest sequencing of familial endometrial cancer patients with this particular haplotype. We added the following text to discussion, page 11, line 318-321:

“Possible European founders were suggested at several loci. These regions would be of special interest for further studies. To find the actual underlying risk causing variant, sequencing in the haplotype regions of familial EC cases would be ideal.”

We also added the following text to conclusion, page 15, line 427-429:

“The finding of possible European founder regions is of special interest and future sequencing of familial EC patients could identify the risk variants at these loci.”

**Reviewer´s comment 4:**
The haplotypes in Table 1 are not brought in context in Figure 1. It would be ideal to see the discovery haplotype along with the replication haplotypes in the same figure.

Response 4:

We agree this would be ideal. Unfortunately, there is a disrepancy between variants in the discovery- (Table 1) and the replication analysis (Figure 1). Therefore the haplotypes in Table 1 and Figure 1 are not comparable. Please see below simplifed illustration over a region with yellow-marked variants from the discovery analysis and turquoise-marked variant from the replication analysis of the Swedish cohort.

…TTAAAAATC…CGCT...TGG…GACTTCGCC…C**T**TCTAGCC…CATATAGTT…

To be able to detect founder regions we decided to reanalyze the Swedish cohort in a combined QC together with the replication cohorts, the Belgian and German, to ensure identical markers between all three cohorts. This is why the 2nd Swedish analysis of the same discovery cohort is presented in Figure 1.

Response to Reviewer 2

Thank you for your very thorough review of our paper and your valuable comments.

The authors conducted a genome-wide haplotype analysis for endometrial cancer in the Swedish female population (1,116 cases + 5,021 controls), followed by replication analyses in two relatively small cohorts. This is a revised manuscript with significant findings and improved clarity on the study population and results presentation, according to the previous review. Since I was not involved in the previous review, I have additional comments.

**Reviewer´s comment 1:**

The Results section could benefit from a more extensive expansion as general readers may not have extensive GWAS/haplotype experience. I recommend providing a summary of the discovery analysis, such as the number of windows and haplotypes being tested, and a clear explanation of how novel loci were defined.

**Response 1:**

We added the text to result to clarify, page 7-8, line 195-205:

“To examine the association between a specific combination of close SNPs (a haplotype) and EC risk a two stage GWAS was performed. In the discovery Swedish dataset of 1,116 EC cases and 5,021 controls 332,906 SNPs were analyzed which resulted in 8,315,750 sliding windows. Significant SNPs or haplotypes in a delimited region is defined as one susceptibility locus and typically represented by several haplotypes of different lengths, OR and statistical significance.”

**Reviewer´s comment 2:**

The authors implemented haplotype analysis using PLINK, but it's unclear how they determined the sliding windows. In one window, multiple haplotypes have a frequency above 1%. Did the authors analyze all the haplotypes in a window in the same logistic regression model? If not, what was the reference the author used to compute the odds ratio and p-values?

**Response 2:**
All genotype data from the first SNP at Chr 1 to the last SNP at Chr X were analyzed using a logistic regression model with a sliding window approach, which is descibed in Material and Method. Reference genome panel GRCh37 was applied.

a) We added the following text to material and methods to clarify, page 5, line 140-141:

“The reference genome panel GRCh37 was applied.”

b) We also added the following text to Table 1 legend, page 8, line 218:

“Reference panel GRCh37”

**Reviewer´s comment 3:**

The authors mentioned in the introduction, 'We hypothesized that haplotype analysis could be more powerful than SNP-only analysis in identifying novel EC susceptibility loci with higher risk.' To support this statement, could the authors display the results of the lead variant with the smallest p-value in the same window as the identified novel haplotypes in Table 1? In addition, the column "SNP1-SNP2" in Table 1 is not informative. It would be better to show the start-end genomic positions instead.

**Response 3:**

a) The SNP with lowest p-value in the novel EC loci presented in Table 1 are presented in S25 Table named “SNPs lowest p-value in novel loci”. The Table is referred to in discussion, page 12, line 329. We agree it should be mentioned in result section. We added the following text to result, page 8, line 211-213:

“One significant SNP was identified on 16q24.3 (OR 1.58, p 3.36x10^-8^) (Table 1, S25 Table and S4 Fig). For the other novel loci, the lowest p-value for one SNP ranged from 3.8x10^-6^ to 0.19 (S25 Table).”

b) We added genomic position BP1-BP2 to Table 1.

**Reviewer´s comment 4:**

I find it surprising that the 15 discovered haplotypes have a high replication rate, considering their relatively low frequencies and small replication cohorts. Their replication analysis wasn't limited to the originally discovered haplotypes, and the replicated haplotypes imply successful replication of the same region but not the discovered risk haplotype. I would expect that a region would replicate as long as it contains at least one variant with a marginally significant effect (i.e., P<0.05). The authors should address this issue in their discussion.

**Response 4:**

The discrepancy in markers between the discovery and replication analysis and the small replication sample sizes limited our ability to replicate the exact identified haplotypes from the discovery analysis and to achieve statistical significance. Still, the regions showed similar ORs and possible founders were suggested. We have modified the text in discussion, page 10, line 275-285:

“Two European cohorts were available for validation analysis. Both were however small, which limited the ability to achieve statistical significance in this analysis. Still, the finding of haplotypes with positive ORs as in the discovery analysis within the tested regions suggested risk haplotypes at the same loci, and even suggested the possibility of European founders. Identification of potential founder mutations required a new QC including all the three cohorts to involve identical SNPs for comparison. This means that the Swedish replication result was not identical to the discovery result. This was expected, as GWAS is highly dependent on the markers used in the analysis. Importantly, all loci had positive ORs in the replication for all three cohorts. All loci which showed compelling associations in the Swedish discovery cohort did not reach the same level of statistical significance in the replication sets.”

Response to Reviewer 3

Thank you for your very thorough review of our paper and your valuable comments.

The study by Barnekow et al. investigates the association between single nucleotide polymorphisms (SNPs) and haplotypes with endometrial cancer risk using a two-stage haplotype-based Genome-Wide Association Study (GWAS) in patients with invasive endometrial cancer. In a previous publication by the same group, this method successfully identified novel susceptibility loci in other cancer types. This current study reinforces the utility of haplotype analysis not only in understanding evolutionary and domestication processes but also in identifying risk loci associated with higher odds ratios compared to those found using SNP analysis alone.
The findings presented are both compelling and novel. Notably, some positive associations replicate findings from earlier studies, such as the association of the locus at 10q26.3 with endometrial cancer, which has been reported in prior research. This replication adds credibility to the new observations in the manuscript. I support the article's publication with few points addressed.

Thanks, the text and reference 28 (in red) is added to discussion, page 13, line 368-370 (Note, that the new ref 28 is not marked “red” in the “revised manuscript with track changes”, which we cannot explain. We have tried to insert/exclude the reference several times but with the same result):

*“TTC40* at 10q26.3 was found to be amplified in HER2-negative gastric cancer in one study and a nearby suggested tumor suppressor gene at locus 10q26.1, the cancer susceptibility candidate 2 gene (*CASC2*), is commonly lost in EC [27, 28].”

**Reviewer´s comment 1:** Lines 255-259
If the creation of overlapping haplotypes means that the same genetic variants could be included in multiple windows, then it is not quite clear to me how the risk signal is not repeatedly counted. Can this overlap potentially contribute to a biased estimate of the odds ratio because the same genetic risk is reflected across multiple haplotypes? While the identified genes are not entirely random and do provide a foundation for their implication in endometrial cancer, the methodological overlap needs careful consideration to avoid inflating the odds ratios artificially.

**Response 1:**

a) We agree the risk signal is repeatedly counted but only counted once in every assoctation tested. Neither do one risk signal from one haplotype impact the other. Methodological bias should therefore not be a problem. What is of importance when it comes to repeatedly counted risk signals, is the interpretation of significant haplotypes in the same region. Haplotypes of various lengths in a delimited region represent the same genetic risk and should therefore be reported as ONE risk locus, in Table 1 represented of the haplotype with lowest p-value in the loci. We added the text (in red) to discussion to clarify, page 12, line 342-344.

“We assumed that the various haplotypes in each delimited region (S1-S23 Tables) reflect the same genetic risk and thus could represent one risk locus.”

b) The association analysis was performed from the first SNP on chromosome 1 to the last SNP on chromosome X without any previous selection. Moreover, the GWAS method and the Oncochip used is a well established method for genetical studies. We agree the Oncochip is not randomly distributed (more densed covered in regions of cancer interest) but the haplotype method would not be more sensitive than SNP GWAS to methodological issues.

**Reviewer´s comment 2:** Lines 221-234

It was mentioned that the Swedish replication cohort was not identical to the discovery cohort, leading to challenges in replicating some of the discovered loci, and that this discrepancy likely arises from differences in the markers used between the two cohorts. Have you considered adjusting the parameters of the combined QC process to see if the replication results might align more closely with the discovery findings? Moreover, do you think that if identical markers had been included in both the discovery and replication cohorts, it would have been possible to replicate most, if not all, of the loci?

**Response 2:**

a) We agree, GWAS result is highly dependeant on which markers are included in the analysis. Therefore it was necessary to replicate the Swedish samples using the same markers as was used in the German and Belgian samples in ECAC. The small difference in number of the Swedish samples in the two analyses is not expected to have an impact on the results. In contrast, analysis with different markers will result in different identified haplotypes, even on the exact same datasets. We added the text (in red) to discussion to clarify, page 10-11, line 285-310:

“Nevertheless, this could be explained by discrepancies between markers in the discovery and the replication analyses. Using different markers in two GWAS conducted on the same data set may not identify the same haplotypes.”

To illustrate the effect please see the simplified example below. The red T is the underlying unknown causal variant. Two separate haplotype analysis are performed with different markers included. In analysis 1 the pink-marked TCTA haplotype showed the lowest (and significant) p-value. In analysis 2 the yellow-marked ATCG haplotype showed the lowest (but unsignificant) p-value.

……TTAAAAATCCGCTTGGGACTTCGCCC**T**TCTAGCCCATATATGTCGGTT…….

b) With respect that we may have misunderstood the question regarding adjustment, we don´t see how the QC parameters from the combined QC could be used for adjustment to better align the result. The only QC parameters which is used in the logistic regression model as covariates is the populations stratification parameters C1,C2,C3 and C4.
c) If the replication cohorts had inlcuded identical markers, probably more loci could have been replicated based on the simplified example above demonstrated in a). However, as different populations are included in the discovery and replication cohorts we cannot expect all loci to be replicated due to genetic population heterogeneity. We added the text (in red) to discussion to clarify, page 11, line 288-290:

“Moreover, due to genetic population heterogeneity between the Swedish, German and Belgian cohorts, we cannot expect all loci to be replicated.”

**Reviewer´s comment 3:**

Minor point:
While the manuscript is well written, there are some minor language errors throughout the manuscript. For example, line 229 – “association” – “associations”, line 230 – “statistically” – “statistical”.

**Response 3:**

Thanks for noticing. It has been corrected in the manuscript.
