# Supplementary material for: A Swedish genome-wide haplotype association analysis identifies novel candidate loci associated with endometrial cancer risk
Source: PLoS One. 2025 Mar 18;20(3):e0316086. doi: 10.1371/journal.pone.0316086 (PMC13537468; doi:10.1371/journal.pone.0316086)
Supplement: Supplementary file 10 [file pone.0316086.s034.docx]

Response to Editor

Thank you for your very thorough review of our paper and your valuable comments to improve the manuscript.

**Editor´s comment 1**

Address the reviewer 2 questions about the haplotype analyses.

A) how did you determine the sliding windows?

B) How was the logistic model set up?, as a multifactor analyses or pairwise analyses of one haplotype agains i) all others or ii) the most common haplotypes? Provide references for these methods.

**Response 1**

The reviewer 2 questions is adressed, please see reviewer 2, response 1.

A) All slidning windows 1-25 from the first SNP on Chr 1 to the last SNP on Chr X were analyzed, as illustrated below for window 1-4. The reason for choosing windows up to 25 was to cover big haplotypes in regions even where the Oncochip is densely covered. The sliding window method is used to try to delimit the region of interest from the first to last the SNP.

…….. up to window 25

B) All haplotypes, with a haplotype frequency above 1%, were tested separately. Each haplotype/SNP was tested againt all others (with a frequency above 1%, excluding the haplotype of interest). We added the text to Material and method and a web site reference [18] together with the previous used reference 16, page 5, line 127-130.

“The default setting of minor haplotype frequency 0.01 in PLINK v.1.07 was applied. This entails that each haplotype or SNP with a frequency above 1% was tested individually against all other SNPs/haplotypes with a frequencies above this threshold [16, 18].”

**Editor´s comment 2**

Clarify the description of the replication. The Swedish cohort (1,139 cases; 6,080 controls) shown in Figure 1, is that a true replication cohort or a subset of the discovery cohort? The latter right?? Wording and descriptions about that are ambiguous, because the figures says the pattern is replicated in the swedish set (but this is not a TRUE replication set). Check this point in main text also!

**Response 2**

We agree, it is not a true replication. The Figure 1 is corrected. All “yes” in the replication column regarding the Swedish samples has been removed.

**Editor´s comment 3**

Also in Figure 1. Why does the 16q24.3 haplotype contains no SNPs?

**Response 3**

Unfotunately, the SNP at 16q24.3 was sorted out during the QC of the replication sets. Therefore this SNP, detected in the discovery analysis, was not analyzed in the replication analysis. This is described in discussion, page 12, line 309-310. We added the text to result, page 9, line 212-214,to clarify how this issue was adressed and presented in Figure 1.

“The significant SNP at 16q24.3 was sorted out during the QC for the replication analysis. Therefore the haplotype with a SNP before and after is presented for comparison in the replication analysis.”

We also added the text (in red) to figure 1 legend to clarify, page 10, line 241:

“The discovery SNP on 16q24.3 (marked with a box) was not analysed in the replication analysis but situated between the two SNPs in the replication haplotype.”

**Editor´s comment 4**

Supplemental figure 1 does not have any axes or units assigned to them. The accompanying legend is not helpful, colours are not described in legend nor on figure. Check all other supplemental figures to see if they suffer same problems and fix accordingly.

**Response 4**

Thanks, the axes have been marked and the colours explained in the legend.

**Editor´s comment 5**

The European founder idea has expanded in this version, without much data being provided to support it. Either tone down (remove from abstract and conclusion) or provide better data or arguments for it.

**Response 5**

The European founder idea has been toned down. In the abstract the word founder is mentioned only in the perspective that this study motivates further studies *to search for* founder haplotypes and in conlusion the founder idea has been removed.

**Editor´s comment 6**

There is some major fault with the WORD on figures/tables and their legends Adjust all figures (supplemental also) and tables.

**Response 6**

- The word format in Figure 1 is converted to “Arial” instead of “Calibri”.
- All “this is Figure 1 title/this is Figure 1 legend” has been removed from figures/tables/legends (supplemental also). Thanks for noticing.

**Minor points**
**Editor´s comment 7**

Line 72.
Wording of new sentence is clonky. “Thus, an identified unique risk haplotype defines the borders of a DNA region holding an assumed risk variant between the first and last marker, most likely not any of the included markers.” Please reword.

**Response 7**

Thanks, the text in Introduction is reworded, page 3, line 74-75:

“Thus, identified unique risk haplotypes define the borders of a DNA region holding an assumed risk variant between the first and last marker”

**Editor´s comment 8**

Line 151
Reword this sentence. Remove “even”, and add a numbers instead of “slightly”.
“Swedish cohort from the discovery analysis was used even in the replication stage, but with slightly more cases and controls included”

**Response 8**

Thanks, the text in Material and method is reworded and the numbers (in red) added, page 6, line 155-157:

“The same Swedish cohort from the discovery analysis was used in the replication stage, but with 6 new cases and 1,059 new controls included (1,139 cases and 6,080 controls).”

**Editor´s comment 9**

Line 213.
Add details “and the Swedish cohort.” To “and the enlarged Swedish cohort (with XX new cases and YY new controls)”

**Response 9**

Thanks, the text (in red) is added to Results, page 9, line 216-218:

“After QC, a separate haplotype analysis with a window size of 1-25 was performed in the two replication cohorts and the enlarged Swedish cohort (with 6 new cases and 1,059 new controls).”

**Editor´s comment 10**

Line 248.
Reword “Still, the finding of haplotypes with positive ORs as in the discovery analysis within the tested regions suggested risk haplotypes at the same loci,”
Maybe “Still, the fact that the haplotypes consistently had positive ORs (same as in the discovery analysis), suggested risk alleles sit within these haplotypes,”

**Response 10**

Thanks, the text in Discussion is reworded (in red), page 10, line 254-256:

“Nonetheless, the consistent presence of positive ORs for the haplotypes, similar to those observed in the discovery analysis, indicated that risk alleles are likely located within these haplotypes and even suggested the possibility of European founders.”

**Editor´s comment 11**

Line 276
“Possible European founders were suggested at several loci.” This claim is not supported by any reference to data. Im OK with it being toned down and just mentioned once. (and not in abstract or conclusion unless better supported and argued).

**Response 11**

Please see response 5. The above text in Discussion has been toned down, page 11, line 278-279:

“Further genotyping studies could search for founder haplotypes”

**Editor´s comment 12**

Line 592.
What are “Complementary rs-numbers“? Reword.

**Response 12**

Thanks for noticing. The word “complementary” is removed, page 22, line 639.

**Editor´s comment 13**

Line 602
Why “a” for Swedish, and German, but not Belgian cohorts?

**Response 13**

Thanks for noticing. We have added the text (in red), page 23, line 657:

“……in a Swedish, a Belgian and a German cohort.”

Response to Reviewer 1

The revised manuscript reads nicely and the authors have addressed the major comments and concerns. The topic is important and will be good addition to haplotype based studies. I support the publication of this manuscript.

Thank you for your thorough review of our paper and previous valuable comments.

Response to Reviewer 2

Thank you for your thorough review of our paper and your valuable comments.

The revised manuscript has addressed my previous comments, except for the second comment: “The authors implemented haplotype analysis using PLINK, but it's unclear how they determined the sliding windows. In one window, multiple haplotypes have a frequency above 1%.

**Reviewer´s comment 1**

Did the authors analyze all the haplotypes in a window in the same logistic regression model? If not, what was the reference the authors used to compute the odds ratio and p-values?” My comment demanded clarification on the setup of the logistic model, not the genome build of their genotype data. Assuming five haplotypes were found in a window with a frequency above 1%, did the authors include the five haplotypes in the same model or test each separately? If the former, the author compared haplotypes against the reference, which included all rare haplotypes with a frequency below 1%. If the latter, the reference in the model would be all haplotypes in the window, excluding the haplotype of interest.

**Response 1**

We apologise for the misunderstanding regarding reference. All haplotypes, with a haplotype frequency above 1%, were tested separately. Each haplotype was tested againt all others (with a frequency above 1%, excluding the haplotype of interest). We added the text to Material and method, page 5, line 127-130.

“The default setting of minor haplotype frequency 0.01 in PLINK v.1.07 was applied. This entails that each haplotype or SNP with a frequency above 1% was tested individually against all other SNPs/haplotypes with a frequencies above this threshold [16, 18].”

Response to Reviewer 3

I appreciate the careful thought you have given to addressing these important points from the reviewers. The additional clarifications you have made ensure that the methodological choices and their implications are clearly understood by the readers.

Thank you for your thorough review of our paper and previous valuable comments.
