# Supplementary material for: A Swedish genome-wide haplotype association analysis identifies novel candidate loci associated with endometrial cancer risk
Source: PLoS One. 2025 Mar 18;20(3):e0316086. doi: 10.1371/journal.pone.0316086 (PMC13537468; doi:10.1371/journal.pone.0316086)
Supplement: Peer review history

# Author comment: Author response to Decision Letter 0

**DOI:** 10.1371/journal.pone.0316086.r001

10 May 2024

Attachment Submitted filename: Response to Reviewer 2 Comments.docx

# Peer review: Decision Letter 0

**DOI:** 10.1371/journal.pone.0316086.r002

**Authors:** Arnar Palsson

9 Sep 2024

PONE-D-24-18312A Swedish genome-wide haplotype association analysis identifies novel candidate loci associated with endometrial cancer riskPLOS ONE

Dear Dr. Barnekow,

Thank you for submitting your manuscript to PLOS ONE. After careful consideration, we feel that it has merit but does not fully meet PLOS ONE’s publication criteria as it currently stands. Therefore, we invite you to submit a revised version of the manuscript that addresses the points raised during the review process.

Please submit your revised manuscript by Oct 24 2024 11:59PM. If you will need more time than this to complete your revisions, please reply to this message or contact the journal office at plosone@plos.org . When you're ready to submit your revision, log on to https://www.editorialmanager.com/pone/ and select the 'Submissions Needing Revision' folder to locate your manuscript file.

Please include the following items when submitting your revised manuscript:

- A rebuttal letter that responds to each point raised by the academic editor and reviewer(s). You should upload this letter as a separate file labeled 'Response to Reviewers'.
- A marked-up copy of your manuscript that highlights changes made to the original version. You should upload this as a separate file labeled 'Revised Manuscript with Track Changes'.
- An unmarked version of your revised paper without tracked changes. You should upload this as a separate file labeled 'Manuscript'.

If you would like to make changes to your financial disclosure, please include your updated statement in your cover letter. Guidelines for resubmitting your figure files are available below the reviewer comments at the end of this letter.

If applicable, we recommend that you deposit your laboratory protocols in protocols.io to enhance the reproducibility of your results. Protocols.io assigns your protocol its own identifier (DOI) so that it can be cited independently in the future. For instructions see: https://journals.plos.org/plosone/s/submission-guidelines#loc-laboratory-protocols . Additionally, PLOS ONE offers an option for publishing peer-reviewed Lab Protocol articles, which describe protocols hosted on protocols.io. Read more information on sharing protocols at https://plos.org/protocols?utm_medium=editorial-email&utm_source=authorletters&utm_campaign=protocols .

We look forward to receiving your revised manuscript.

Kind regards,

Arnar Palsson, Ph.D.

Academic Editor

PLOS ONE

Journal Requirements:

When submitting your revision, we need you to address these additional requirements.

1. Please ensure that your manuscript meets PLOS ONE's style requirements, including those for file naming. The PLOS ONE style templates can be found at 

https://journals.plos.org/plosone/s/file?id=wjVg/PLOSOne_formatting_sample_main_body.pdf and 

https://journals.plos.org/plosone/s/file?id=ba62/PLOSOne_formatting_sample_title_authors_affiliations.pdf

2. We note that the grant information you provided in the ‘Funding Information’ and ‘Financial Disclosure’ sections do not match. 

When you resubmit, please ensure that you provide the correct grant numbers for the awards you received for your study in the ‘Funding Information’ section.

3. In the online submission form, you indicated that "The data generated in this study are publicly available at Zenodo ( DOI

10.5281/zenodo.10633111); S1-23 Tables. The entire data, including individual and

personal identifying data, is not publicly available due to regulations, privacy, or ethical restrictions. The data supporting the findings of this study are available from the

corresponding authors upon reasonable request."

All PLOS journals now require all data underlying the findings described in their manuscript to be freely available to other researchers, either 1. In a public repository, 2. Within the manuscript itself, or 3. Uploaded as supplementary information.

This policy applies to all data except where public deposition would breach compliance with the protocol approved by your research ethics board. If your data cannot be made publicly available for ethical or legal reasons (e.g., public availability would compromise patient privacy), please explain your reasons on resubmission and your exemption request will be escalated for approval. 

4. Please review your reference list to ensure that it is complete and correct. If you have cited papers that have been retracted, please include the rationale for doing so in the manuscript text, or remove these references and replace them with relevant current references. Any changes to the reference list should be mentioned in the rebuttal letter that accompanies your revised manuscript. If you need to cite a retracted article, indicate the article’s retracted status in the References list and also include a citation and full reference for the retraction notice.

Additional Editor Comments:

The manuscript is interesting and tackles a very important disorder. The haplotype focus is also an advanced topic of broader relevance. The reviewers were quite enthusiastic, but raised a few points that need clarifying mainly. No need for more analyses or additional work beyond adjustments to text and visuals.

The major points are:

1. We recommend providing a summary of the discovery analysis, such as the number of windows and haplotypes being tested (rev2)

2. Clarify how corrections for multiple testing were handled in replication cohorts (rev1), see also concern from rev3 about “overlapping/repeated” signals. Also, are the p values and OR´s in figure 1 from pairwise tests of haplotypes or what??

3. Rev 3 raised a concern about the replication cohort. Please clarify.

4. Figure 1 legend should include the sample sizes for each of the cohorts (rev1)

5. It is worthwhile to discuss also the potential reasons for haplotype associations. Is it due to the genetic influence from multiple single markers or combinatorial effects of many markers?

The reviewers add several good pointers and suggestions. Note, on comment 3 from rev 2. We suggest adding genomic coordinates (but not dropping the SNP ids, they are helpful).

[Note: HTML markup is below. Please do not edit.]

Reviewers' comments:

Reviewer's Responses to Questions

**Comments to the Author**

1. Is the manuscript technically sound, and do the data support the conclusions?

The manuscript must describe a technically sound piece of scientific research with data that supports the conclusions. Experiments must have been conducted rigorously, with appropriate controls, replication, and sample sizes. The conclusions must be drawn appropriately based on the data presented. 

Reviewer #1: No

Reviewer #2: Yes

Reviewer #3: Yes

**********

2. Has the statistical analysis been performed appropriately and rigorously? 

Reviewer #1: No

Reviewer #2: Yes

Reviewer #3: I Don't Know

**********

3. Have the authors made all data underlying the findings in their manuscript fully available?

The PLOS Data policy requires authors to make all data underlying the findings described in their manuscript fully available without restriction, with rare exception (please refer to the Data Availability Statement in the manuscript PDF file). The data should be provided as part of the manuscript or its supporting information, or deposited to a public repository. For example, in addition to summary statistics, the data points behind means, medians and variance measures should be available. If there are restrictions on publicly sharing data—e.g. participant privacy or use of data from a third party—those must be specified.

Reviewer #1: Yes

Reviewer #2: Yes

Reviewer #3: Yes

**********

4. Is the manuscript presented in an intelligible fashion and written in standard English?

PLOS ONE does not copyedit accepted manuscripts, so the language in submitted articles must be clear, correct, and unambiguous. Any typographical or grammatical errors should be corrected at revision, so please note any specific errors here.

Reviewer #1: Yes

Reviewer #2: Yes

Reviewer #3: Yes

**********

5. Review Comments to the Author

Please use the space provided to explain your answers to the questions above. You may also include additional comments for the author, including concerns about dual publication, research ethics, or publication ethics. (Please upload your review as an attachment if it exceeds 20,000 characters)

Reviewer #1: Originality: It is perhaps obvious that haplotypes contain more information than individual SNPs for GWAS studies (as the authors and other has shown in prior studies)

Scientific Quality: Careful attention to details, adequate sample sizes, important inclusion of a relevant replication group

General Comment: The paper is well written

Comment 1:

The manuscript list three replication cohorts Swedish, Belgian and German. It is not clear what samples are part of the Swedish replication cohort. Figure 1 as well lists 3 datasets as replication cohorts. The legend should include the sample sizes for each of the cohorts.

Comment 2:

The authors suggest a p<0.05 as significance threshold for the replication cohorts. However, for each discovered haplotypes of interest the study tests multiple distinct haplotypes in the replication cohorts. In such scenario there should be multiple testing correction performed.

Comment 3:

Only 4p15.31 and 12q13.11 loci share a common haplotype background in the replication cohorts as per figure 1. Possibly imputation should be performed for these regions to identify risk marker.

Comment 4:

The haplotypes in Table 1 are not brought in context in Figure 1. It would be ideal to see the discovery haplotype along with the replication haplotypes in the same figure.

Reviewer #2: The authors conducted a genome-wide haplotype analysis for endometrial cancer in the Swedish female population (1,116 cases + 5,021 controls), followed by replication analyses in two relatively small cohorts. This is a revised manuscript with significant findings and improved clarity on the study population and results presentation, according to the previous review. Since I was not involved in the previous review, I have additional comments.

1. The Results section could benefit from a more extensive expansion as general readers may not have extensive GWAS/haplotype experience. I recommend providing a summary of the discovery analysis, such as the number of windows and haplotypes being tested, and a clear explanation of how novel loci were defined.

2. The authors implemented haplotype analysis using PLINK, but it's unclear how they determined the sliding windows. In one window, multiple haplotypes have a frequency above 1%. Did the authors analyze all the haplotypes in a window in the same logistic regression model? If not, what was the reference the author used to compute the odds ratio and p-values?

3. The authors mentioned in the introduction, 'We hypothesized that haplotype analysis could be more powerful than SNP-only analysis in identifying novel EC susceptibility loci with higher risk.' To support this statement, could the authors display the results of the lead variant with the smallest p-value in the same window as the identified novel haplotypes in Table 1? In addition, the column "SNP1-SNP2" in Table 1 is not informative. It would be better to show the start-end genomic positions instead.

4. I find it surprising that the 15 discovered haplotypes have a high replication rate, considering their relatively low frequencies and small replication cohorts. Their replication analysis wasn't limited to the originally discovered haplotypes, and the replicated haplotypes imply successful replication of the same region but not the discovered risk haplotype. I would expect that a region would replicate as long as it contains at least one variant with a marginally significant effect (i.e., P<0.05). The authors should address this issue in their discussion.

Reviewer #3: The study by Barnekow et al. investigates the association between single nucleotide polymorphisms (SNPs) and haplotypes with endometrial cancer risk using a two-stage haplotype-based Genome-Wide Association Study (GWAS) in patients with invasive endometrial cancer. In a previous publication by the same group, this method successfully identified novel susceptibility loci in other cancer types. This current study reinforces the utility of haplotype analysis not only in understanding evolutionary and domestication processes but also in identifying risk loci associated with higher odds ratios compared to those found using SNP analysis alone.

The findings presented are both compelling and novel. Notably, some positive associations replicate findings from earlier studies, such as the association of the locus at 10q26.3 with endometrial cancer, which has been reported in prior research. This replication adds credibility to the new observations in the manuscript. I support the article's publication with few points addressed.

1) Lines 255-259

If the creation of overlapping haplotypes means that the same genetic variants could be included in multiple windows, then it is not quite clear to me how the risk signal is not repeatedly counted. Can this overlap potentially contribute to a biased estimate of the odds ratio because the same genetic risk is reflected across multiple haplotypes? While the identified genes are not entirely random and do provide a foundation for their implication in endometrial cancer, the methodological overlap needs careful consideration to avoid inflating the odds ratios artificially.

2) Lines 221-234

It was mentioned that the Swedish replication cohort was not identical to the discovery cohort, leading to challenges in replicating some of the discovered loci, and that this discrepancy likely arises from differences in the markers used between the two cohorts. Have you considered adjusting the parameters of the combined QC process to see if the replication results might align more closely with the discovery findings? Moreover, do you think that if identical markers had been included in both the discovery and replication cohorts, it would have been possible to replicate most, if not all, of the loci?

Minor point:

While the manuscript is well written, there are some minor language errors throughout the manuscript. For example, line 229 – “association” – “associations”, line 230 – “statistically” – “statistical”.

**********

6. PLOS authors have the option to publish the peer review history of their article (what does this mean? ). If published, this will include your full peer review and any attached files.

If you choose “no”, your identity will remain anonymous but your review may still be made public.

**Do you want your identity to be public for this peer review?** For information about this choice, including consent withdrawal, please see our Privacy Policy .

Reviewer #1: No

Reviewer #2: **Yes: ** Fan Wang

Reviewer #3: No

**********

[NOTE: If reviewer comments were submitted as an attachment file, they will be attached to this email and accessible via the submission site. Please log into your account, locate the manuscript record, and check for the action link "View Attachments". If this link does not appear, there are no attachment files.]

While revising your submission, please upload your figure files to the Preflight Analysis and Conversion Engine (PACE) digital diagnostic tool, https://pacev2.apexcovantage.com/ . PACE helps ensure that figures meet PLOS requirements. To use PACE, you must first register as a user. Registration is free. Then, login and navigate to the UPLOAD tab, where you will find detailed instructions on how to use the tool. If you encounter any issues or have any questions when using PACE, please email PLOS at figures@plos.org . Please note that Supporting Information files do not need this step.

# Author comment: Author response to Decision Letter 0

**DOI:** 10.1371/journal.pone.0316086.r003

16 Oct 2024

Thank you for your very thorough review of our paper and your valuable comments.

A new version of the manuscript (with and without track changes) along with the rebuttal letter are now uploaded. Please note that there is a discrepancy between lines in the “Revised Manuscript with Track Changes” and “Manuscript”. The pages and lines in responses to editor and reviewers refers to the “Revised Manuscript with Track Changes”.

The Figure 1 is remodelled in the PACE-program as requested. As changes are not tracked in Figure 1, we will inform you that “NA” and “no” in the replication and founder column i excluded to increase readability.

The replication analysis is no available at the public repository (S26 Table), therefore the previous S 26 Table is no called S 27 Table.

The reference 28 is added to the mansucript.

Attachment Submitted filename: Rebuttal letter.docx

# Peer review: Decision Letter 1

**DOI:** 10.1371/journal.pone.0316086.r004

**Authors:** Arnar Palsson

6 Nov 2024

PONE-D-24-18312R1A Swedish genome-wide haplotype association analysis identifies novel candidate loci associated with endometrial cancer riskPLOS ONE

Dear Dr. Barnekow,

Thank you for submitting your manuscript to PLOS ONE. After careful consideration, we feel that it has merit but does not fully meet PLOS ONE’s publication criteria as it currently stands. Therefore, we invite you to submit a revised version of the manuscript that addresses the points raised during the review process.

Address the reviewer 2 questions about the haplotype analyses. A) how did you determine the sliding windows? B) How was the logistic model set up?, as a multifactor analyses or pairwise analyses of one haplotype agains i) all others or ii) the most common haplotypes? Provide references for these methods.

Clarify the description of the replication. The Swedish cohort (1,139 cases; 6,080 controls) shown in Figure 1, is that a true replication cohort or a subset of the discovery cohort? The latter right?? Wording and descriptions about that are ambiguous, because the figures says the pattern is replicated in the swedish set (but this is not a TRUE replication set). Check this point in main text also!

Also in Figure 1. Why does the 16q24.3 haplotype contains no SNPs?

Supplemental figure 1 does not have any axes or units assigned to them. The accompanying legend is not helpful, colours are not described in legend nor on figure. Check all other supplemental figures to see if they suffer same problems and fix accordingly.

The European founder idea has expanded in this version, without much data being provided to support it. Either tone down (remove from abstract and conclusion) or provide better data or arguments for it.

There is some major fault with the WORD on figures/tables and their legends Adjust all figures (supplemental also) and tables.

Please submit your revised manuscript by Dec 21 2024 11:59PM. If you will need more time than this to complete your revisions, please reply to this message or contact the journal office at plosone@plos.org . When you're ready to submit your revision, log on to https://www.editorialmanager.com/pone/ and select the 'Submissions Needing Revision' folder to locate your manuscript file.

Please include the following items when submitting your revised manuscript:

- A rebuttal letter that responds to each point raised by the academic editor and reviewer(s). You should upload this letter as a separate file labeled 'Response to Reviewers'.
- A marked-up copy of your manuscript that highlights changes made to the original version. You should upload this as a separate file labeled 'Revised Manuscript with Track Changes'.
- An unmarked version of your revised paper without tracked changes. You should upload this as a separate file labeled 'Manuscript'.

If you would like to make changes to your financial disclosure, please include your updated statement in your cover letter. Guidelines for resubmitting your figure files are available below the reviewer comments at the end of this letter.

If applicable, we recommend that you deposit your laboratory protocols in protocols.io to enhance the reproducibility of your results. Protocols.io assigns your protocol its own identifier (DOI) so that it can be cited independently in the future. For instructions see: https://journals.plos.org/plosone/s/submission-guidelines#loc-laboratory-protocols . Additionally, PLOS ONE offers an option for publishing peer-reviewed Lab Protocol articles, which describe protocols hosted on protocols.io. Read more information on sharing protocols at https://plos.org/protocols?utm_medium=editorial-email&utm_source=authorletters&utm_campaign=protocols .

We look forward to receiving your revised manuscript.

Kind regards,

Arnar Palsson, Ph.D.

Academic Editor

PLOS ONE

Journal Requirements:

Please review your reference list to ensure that it is complete and correct. If you have cited papers that have been retracted, please include the rationale for doing so in the manuscript text, or remove these references and replace them with relevant current references. Any changes to the reference list should be mentioned in the rebuttal letter that accompanies your revised manuscript. If you need to cite a retracted article, indicate the article’s retracted status in the References list and also include a citation and full reference for the retraction notice.

Additional Editor Comments:

Address the reviewer 2 questions about the haplotype analyses. A) how did you determine the sliding windows? B) How was the logistic model set up?, as a multifactor analyses or pairwise analyses of one haplotype agains i) all others or ii) the most common haplotypes? Provide references for these methods.

Clarify the description of the replication. The Swedish cohort (1,139 cases; 6,080 controls) shown in Figure 1, is that a true replication cohort or a subset of the discovery cohort? The latter right?? Wording and descriptions about that are ambiguous, because the figures says the pattern is replicated in the swedish set (but this is not a TRUE replication set). Check this point in main text also!

Also in Figure 1. Why does the 16q24.3 haplotype contains no SNPs?

Supplemental figure 1 does not have any axes or units assigned to them. The accompanying legend is not helpful, colours are not described in legend nor on figure. Check all other supplemental figures to see if they suffer same problems and fix accordingly.

The European founder idea has expanded in this version, without much data being provided to support it. Either tone down (remove from abstract and conclusion) or provide better data or arguments for it.

There is some major fault with the WORD on figures/tables and their legends Adjust all figures (supplemental also) and tables.

Minor points

Line 72.

Wording of new sentence is clonky. “Thus, an identified unique risk haplotype defines the

borders of a DNA region holding an assumed risk variant between the first and last marker,

most likely not any of the included markers.” Please reword.

Line 151

Reword this sentence. Remove “even”, and add a numbers instead of “slightly”.

“Swedish cohort from the discovery analysis was used even in the replication stage, but with

slightly more cases and controls included”

Line 213.

Add details “and the Swedish cohort.” To “and the enlarged Swedish cohort (with XX new cases and YY new controls)”

Line 248.

Reword “Still, the finding of haplotypes with positive ORs as in the discovery analysis within the tested regions suggested risk haplotypes at the same loci,”

Maybe “Still, the fact that the haplotypes consistently had positive ORs (same as in the discovery analysis),

suggested risk alleles sit within these haplotypes,”

Line 276

“Possible European founders were suggested at several loci.” This claim is not supported by any reference to data. Im OK with it being toned down and just mentioned once. (and not in abstract or conclusion unless better supported and argued).

Line 592.

What are “Complementary rs-numbers“? Reword.

Line 602

Why “a” for Swedish, and German, but not Belgian cohorts?

[Note: HTML markup is below. Please do not edit.]

Reviewers' comments:

Reviewer's Responses to Questions

**Comments to the Author**

1. If the authors have adequately addressed your comments raised in a previous round of review and you feel that this manuscript is now acceptable for publication, you may indicate that here to bypass the “Comments to the Author” section, enter your conflict of interest statement in the “Confidential to Editor” section, and submit your "Accept" recommendation.

Reviewer #1: All comments have been addressed

Reviewer #2: (No Response)

Reviewer #3: All comments have been addressed

**********

2. Is the manuscript technically sound, and do the data support the conclusions?

The manuscript must describe a technically sound piece of scientific research with data that supports the conclusions. Experiments must have been conducted rigorously, with appropriate controls, replication, and sample sizes. The conclusions must be drawn appropriately based on the data presented. 

Reviewer #1: Yes

Reviewer #2: Yes

Reviewer #3: Yes

**********

3. Has the statistical analysis been performed appropriately and rigorously? 

Reviewer #1: Yes

Reviewer #2: Yes

Reviewer #3: Yes

**********

4. Have the authors made all data underlying the findings in their manuscript fully available?

The PLOS Data policy requires authors to make all data underlying the findings described in their manuscript fully available without restriction, with rare exception (please refer to the Data Availability Statement in the manuscript PDF file). The data should be provided as part of the manuscript or its supporting information, or deposited to a public repository. For example, in addition to summary statistics, the data points behind means, medians and variance measures should be available. If there are restrictions on publicly sharing data—e.g. participant privacy or use of data from a third party—those must be specified.

Reviewer #1: Yes

Reviewer #2: Yes

Reviewer #3: Yes

**********

5. Is the manuscript presented in an intelligible fashion and written in standard English?

PLOS ONE does not copyedit accepted manuscripts, so the language in submitted articles must be clear, correct, and unambiguous. Any typographical or grammatical errors should be corrected at revision, so please note any specific errors here.

Reviewer #1: Yes

Reviewer #2: Yes

Reviewer #3: Yes

**********

6. Review Comments to the Author

Please use the space provided to explain your answers to the questions above. You may also include additional comments for the author, including concerns about dual publication, research ethics, or publication ethics. (Please upload your review as an attachment if it exceeds 20,000 characters)

Reviewer #1: The revised manuscript reads nicely and the authors have addressed the major comments and concerns. The topic is important and will be good addition to haplotype based studies. I support the publication of this manuscript.

Reviewer #2: The revised manuscript has addressed my previous comments, except for the second comment: “The authors implemented haplotype analysis using PLINK, but it's unclear how they determined the sliding windows. In one window, multiple haplotypes have a frequency above 1%. Did the authors analyze all the haplotypes in a window in the same logistic regression model? If not, what was the reference the authors used to compute the odds ratio and p-values?”

My comment demanded clarification on the setup of the logistic model, not the genome build of their genotype data. Assuming five haplotypes were found in a window with a frequency above 1%, did the authors include the five haplotypes in the same model or test each separately? If the former, the author compared haplotypes against the reference, which included all rare haplotypes with a frequency below 1%. If the latter, the reference in the model would be all haplotypes in the window, excluding the haplotype of interest.

Reviewer #3: I appreciate the careful thought you have given to addressing these important points from the reviewers. The additional clarifications you have made ensure that the methodological choices and their implications are clearly understood by the readers.

**********

7. PLOS authors have the option to publish the peer review history of their article (what does this mean? ). If published, this will include your full peer review and any attached files.

If you choose “no”, your identity will remain anonymous but your review may still be made public.

**Do you want your identity to be public for this peer review?** For information about this choice, including consent withdrawal, please see our Privacy Policy .

Reviewer #1: No

Reviewer #2: **Yes: ** Fan Wang

Reviewer #3: No

**********

[NOTE: If reviewer comments were submitted as an attachment file, they will be attached to this email and accessible via the submission site. Please log into your account, locate the manuscript record, and check for the action link "View Attachments". If this link does not appear, there are no attachment files.]

While revising your submission, please upload your figure files to the Preflight Analysis and Conversion Engine (PACE) digital diagnostic tool, https://pacev2.apexcovantage.com/ . PACE helps ensure that figures meet PLOS requirements. To use PACE, you must first register as a user. Registration is free. Then, login and navigate to the UPLOAD tab, where you will find detailed instructions on how to use the tool. If you encounter any issues or have any questions when using PACE, please email PLOS at figures@plos.org . Please note that Supporting Information files do not need this step.

# Author comment: Author response to Decision Letter 1

**DOI:** 10.1371/journal.pone.0316086.r005

28 Nov 2024

All comments from Academic editor and reviewer 2 is adressed, please see the uploaded file "Response to reviewers". A new version of "manuscript with track changes", a "manuscrip"t without track changes, a new version of "S1_figure 1" (with marked axes) and "Figure 1" (with changed text style from "calibri" to "arial" and without "yes" in replication column för Swedish samples) is also uploaded.

Attachment Submitted filename: Rebuttal_letter_auresp_2.docx

# Editor report: Decision Letter 2

**DOI:** 10.1371/journal.pone.0316086.r006

**Authors:** Arnar Palsson

6 Dec 2024

A Swedish genome-wide haplotype association analysis identifies novel candidate loci associated with endometrial cancer risk

PONE-D-24-18312R2

Dear Dr. Barnekow,

We’re pleased to inform you that your manuscript has been judged scientifically suitable for publication and will be formally accepted for publication once it meets all outstanding technical requirements.

Within one week, you’ll receive an e-mail detailing the required amendments. When these have been addressed, you’ll receive a formal acceptance letter and your manuscript will be scheduled for publication.

An invoice will be generated when your article is formally accepted. Please note, if your institution has a publishing partnership with PLOS and your article meets the relevant criteria, all or part of your publication costs will be covered. Please make sure your user information is up-to-date by logging into Editorial Manager at Editorial Manager®  and clicking the ‘Update My Information' link at the top of the page. If you have any questions relating to publication charges, please contact our Author Billing department directly at authorbilling@plos.org.

If your institution or institutions have a press office, please notify them about your upcoming paper to help maximize its impact. If they’ll be preparing press materials, please inform our press team as soon as possible -- no later than 48 hours after receiving the formal acceptance. Your manuscript will remain under strict press embargo until 2 pm Eastern Time on the date of publication. For more information, please contact onepress@plos.org.

Kind regards,

Arnar Palsson, Ph.D.

Academic Editor

PLOS ONE

Additional Editor Comments (optional):

Reviewers' comments:

# Editor report: Acceptance letter

**DOI:** 10.1371/journal.pone.0316086.r007

**Authors:** Arnar Palsson

PONE-D-24-18312R2

PLOS ONE

Dear Dr. Barnekow,

I'm pleased to inform you that your manuscript has been deemed suitable for publication in PLOS ONE. Congratulations! Your manuscript is now being handed over to our production team.

At this stage, our production department will prepare your paper for publication. This includes ensuring the following:

* All references, tables, and figures are properly cited

* All relevant supporting information is included in the manuscript submission,

* There are no issues that prevent the paper from being properly typeset

If revisions are needed, the production department will contact you directly to resolve them. If no revisions are needed, you will receive an email when the publication date has been set. At this time, we do not offer pre-publication proofs to authors during production of the accepted work. Please keep in mind that we are working through a large volume of accepted articles, so please give us a few weeks to review your paper and let you know the next and final steps.

Lastly, if your institution or institutions have a press office, please let them know about your upcoming paper now to help maximize its impact. If they'll be preparing press materials, please inform our press team within the next 48 hours. Your manuscript will remain under strict press embargo until 2 pm Eastern Time on the date of publication. For more information, please contact onepress@plos.org.

If we can help with anything else, please email us at customercare@plos.org.

Thank you for submitting your work to PLOS ONE and supporting open access.

Kind regards,

PLOS ONE Editorial Office Staff

on behalf of

Dr. Arnar Palsson

Academic Editor

PLOS ONE
